# Hominin glacial-stage occupation 712,000 to 424,000 years ago at Fordwich Pit, Old Park (Canterbury, UK)

**DOI:** 10.1038/s41559-025-02829-x

**Published:** 2025-09-01

**Authors:** Alastair Key, James Clark, Tobias Lauer, Jennifer Bates, Mark-Jan Sier, Claire Nichols, Carmen Martín-Ramos, Adela Cebeiro, Eleanor Williams, Sunghui Kim, Finn Stileman, Anna Mika, Matthew Pope, David Bridgland, David Redhouse, Michela Leonardi, Geoff M. Smith, Tomos Proffitt

**Affiliations:** 1 Department of Archaeology, University of Cambridge, Cambridge, UK; 2 Terrestrial Sedimentology, Department of Geosciences, Eberhard Karls Universität Tübingen, Tübingen, Germany; 3 Department of Archaeology and Art History, Seoul National University, Seoul, South Korea; 4 Department of Earth Sciences, Faculty of Geosciences, Utrecht University, Utrecht, the Netherlands; 5 CENIEH (National Research Center on Human Evolution), Burgos, Spain; 6 Department of Earth Sciences, University of Oxford, Oxford, UK; 7 Department of Archaeology and Ancient History, University of Leicester, Leicester, UK; 8 Department of Anthropology, New York University, New York, NY USA; 9 Department of Archaeology, Institute of History, Consejo Superior de Investigaciones Científicas (CSIC), Madrid, Spain; 10 Institute of Archaeology, University College London, London, UK; 11 Department of Geography, Durham University, Durham, UK; 12 Department of Zoology, University of Cambridge, Cambridge, UK; 13 Natural History Museum, London, UK; 14 Department of Archaeology, University of Reading, Reading, UK; 15 Interdisciplinary Center for Archaeology and Evolution of Human Behaviour (ICArEHB), Universidade do Algarve, Faro, Portugal

**Keywords:** Archaeology, Biological anthropology

## Abstract

Few high-latitude archaeological contexts are older than marine isotope stage (MIS) 15 and even fewer provide evidence of early human occupation during a glacial period. New discoveries at Old Park, Canterbury (UK), provide evidence of both the oldest accessible artefact-bearing sediment in northern Europe and cold-stage adaptation. Radiometric and palaeomagnetic dating places the earliest suggested occupation of this site between 773 thousand years ago (ka) and 607 ka, with hominin presence inferred during MIS 17–16. Two additional artefact-bearing stratigraphic units, dated to around 542 ka and 437 ka, strongly align with the MIS 14 and 12 cold stages, respectively. The latter unit contains convincing evidence of glacial-stage occupation by Acheulean hominins; fresh, unabraded flakes (including biface-thinning) between clearly defined glacial-aged sediments displaying mixed grassland palaeoenvironmental evidence. An historically collected assemblage of more than 330 handaxes is argued to be derived from both the MIS 17–16 and MIS 12 sediments, providing evidence of the earliest known Acheulean bifaces in northern Europe, and re-occupation by Acheulean populations 200,000 years later. Together, Old Park provides evidence for Lower Palaeolithic hominins reoccupying a location over several mid-Pleistocene MIS cycles, early human presence above 51° latitude during a glacial stage and handaxe production in northern Europe from MIS 17 to 16.

## Abstract (web summary)

The authors report Acheulean hominin occupation of eastern Britain during glacial marine isotope stages 17–16 and again in glacial marine isotope stage 12 via stone tools in sediments dated to 712,000 to 424,000 years ago.

## Main

The ability to survive in harsh and variable environments, including high latitudes, is a hallmark of behavioural flexibility in humans^1^. Hominins first colonized northern Europe during the early Pleistocene but archaeological and fossil evidence of these incursions is rare^2–4^. As a result, little is known about these populations, yet they represent the earliest known human presence at a high latitude and provide an important behavioural and evolutionary comparative perspective for more southerly evidenced groups that include *Homo antecessor*, *Homo erectus* and, later, *Homo heidelbergensis*^5,6^.

Only 6 radiometrically dated Palaeolithic occurrences are known between 960 thousand years ago (ka) and 620 ka in northern Europe^3,7–13^. Only la Noira (marine isotope stage (MIS) 17/16, central France) and Moulin Quignon (MIS 16, northern France) provide evidence of Acheulean bifacially flaked core technologies, and therefore diversity and complexity in material culture, at this early point^3,10^. Furthermore, if la Noira, located at about 47° N latitude, is not included in ‘northern Europe’ definitions (compare refs. ^4,10^), then no pre-MIS 15 sites in this region are easily accessible and readily open to future archaeological endeavours. Indeed, all are located in cliffs^2,7^, embedded under substantial depths of gravel^12^ or within rarely accessible fluvial sediment^10,12^. This includes Moulin Quignon (Somme Valley, France), which dates to 670–650 ka and provides one of the few potential examples of hominin presence in northern Europe during a glacial period^8,10^. Identifying new evidence of hominins in northern Europe before MIS 15 is, therefore, of utmost importance to human origins research in Europe, and to the global understanding of early hominin presence in high latitudes.

From 533 ka to 478 ka (MIS 13) onwards, Lower Palaeolithic evidence is more frequently observed in northern Europe^12,14,15^. Handaxes and scraper technologies become widespread and our understanding of hominin behaviour in high latitudes becomes more detailed, with controlled fire use, organic technologies and diverse lithic reduction processes evidenced^16–19^. These behaviours potentially characterize an expansion of the hominin niche, although evidence of glacial-stage occupation is still largely absent from southern Britain^8,15^.

It is clear that our understanding of hominin presence in northern Europe during the early-to-middle Pleistocene is severely lacking and there are few archaeological sites to provide new findings. We do not know, for example, whether handaxes—and therefore the Acheulean tradition^20^—were widely present above 48° N in MIS 16–17, and potentially even earlier, or whether Moulin Quignon is an exceptional outlier. We do not know whether present site temporal and spatial distributions accurately reflect the first arrival of hominins in this region^4^. Nor do we know whether hominins were frequently present during cold stages (glacial periods), what environmental conditions supported these potential visits or how reliable some inferred instances of this behaviour are^8^. Finally, as a result of challenging taphonomic and geological contexts, we often do not know if hominins repeatedly visited the few sites that are known.

Here, we address these questions by reporting on new excavations, dating, palaeoenvironmental evidence and artefacts from the Chequer’s Wood and Old Park Site of Special Scientific Interest (SSSI) in Canterbury, Kent (UK) (hereafter ‘Old Park’, including ‘Fordwich Pit’).

## Results

**Fig. 1:**
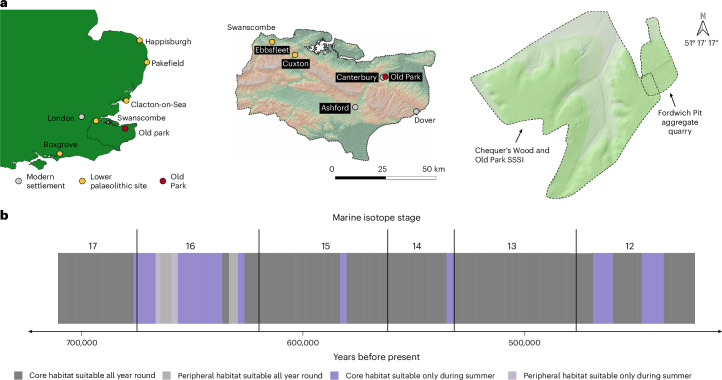
Maps depicting the location of Old Park within Britain, Kent and relative to Fordwich Pit, alongside the environmental suitability of Old Park. **a**, Maps depicting the location of Old Park within Britain (top left), Kent (top centre) and relative to Fordwich Pit (top right). **b**, The environmental suitability of Old Park to support Acheulean hominin populations through MIS 17–12 following Leonardi et al.^42^ (Supplementary Information). These data demonstrate Old Park to have probably been suitable for hominin habitation throughout the year during the majority of MIS 17, 15, 14 and 13, while MIS 16 and 12 are more regularly characterized as only being suitable for seasonal summer habitation, with mean winter temperatures below −5 °C.

Most of Old Park is located on undisturbed Quaternary fluvial deposits derived from the River Stour and backs directly onto the city of Canterbury (Fig. 1). Located at around 40–45 m ordnance datum, Old Park retains some of the highest, and therefore probably oldest, artefact-bearing Quaternary terraces in northern Europe^21,22^. We have observed flake artefacts to be eroding from the highest terraces within Old Park in several locations (Supplementary Fig. 1).

**Fig. 2:**
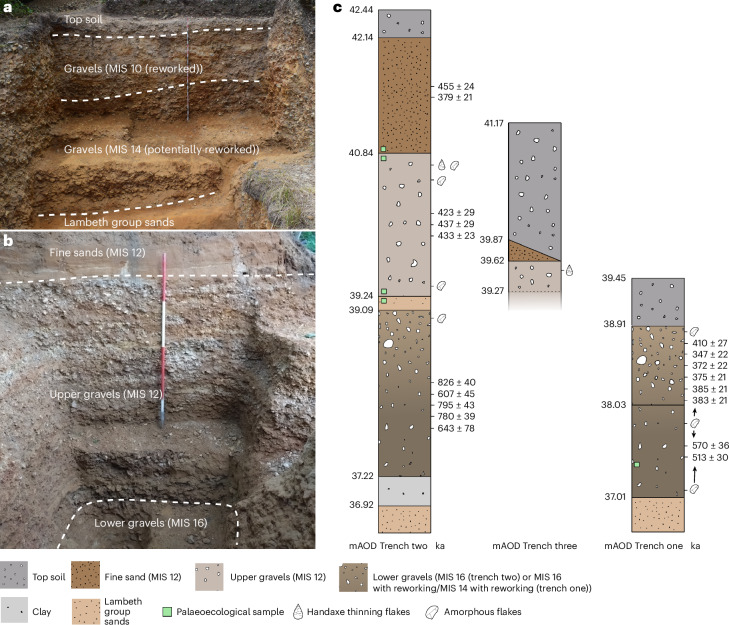
Stratigraphic data from Fordwich Pit, Old Park. **a**,**b**, Trench one (**a**) and trench two (**b**) with dated layers highlighted following ref. ^25^ and this paper. **c**, The location of the palaeoecological, IR-RF samples and artefacts from within the stratigraphy of all three trenches. Note that the base of trench three is faded as we have not yet extended the excavation all the way through the upper gravels. See also Supplementary Fig. 2. mAOD, metres above ordnance datum.

**Fig. 3:**
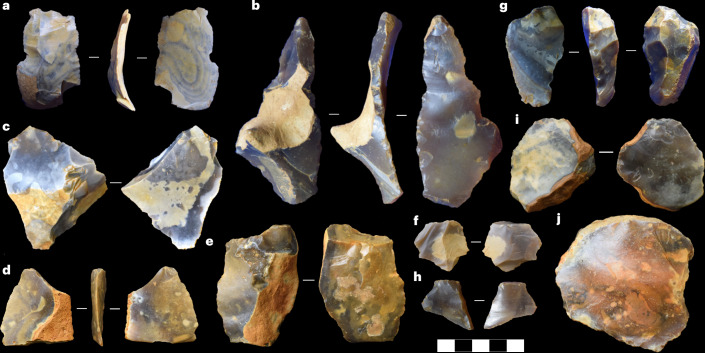
A selection of flake artefacts from Old Park. **a**–**j**, This includes two biface-thinning flakes from the top of the upper gravels (1.5–1.6 m) (**a**,**b**), several fresh flakes from the top of the upper gravels (1.5–1.6 m) (**c**–**f**), one flake from the upper gravels lower limit (3.4–3.5 m) (**g**), one probable flake from the lower gravels (3.6–3.7 m) (**h**) and two surface finds from the western edge of the SSSI (westernmost highlight area in Supplementary Fig. 1) (**i**,**j**). The latter artefacts were required to be left on-site. The scale bar is 5 cm.

**Extended Data Fig. 1:**
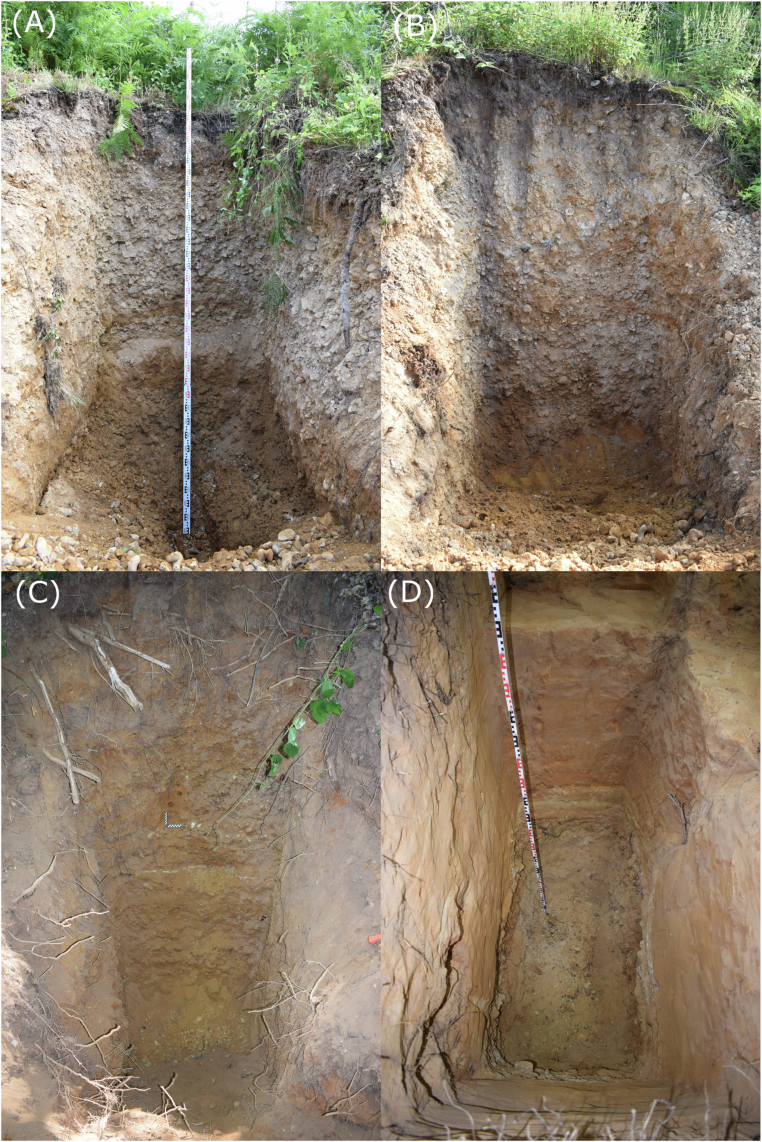
Test trenches and exposures from around the quarry’s edge. A selection of test trenches and exposures from around the quarry’s edge. (**a**–**c**), alongside the opening of Trench Two (**d**). Clearly visible at the bottom of the trench in figure D is the fine sand-to-gravel boundary where the initial sharp flakes were discovered.

**Extended Data Fig. 2:**
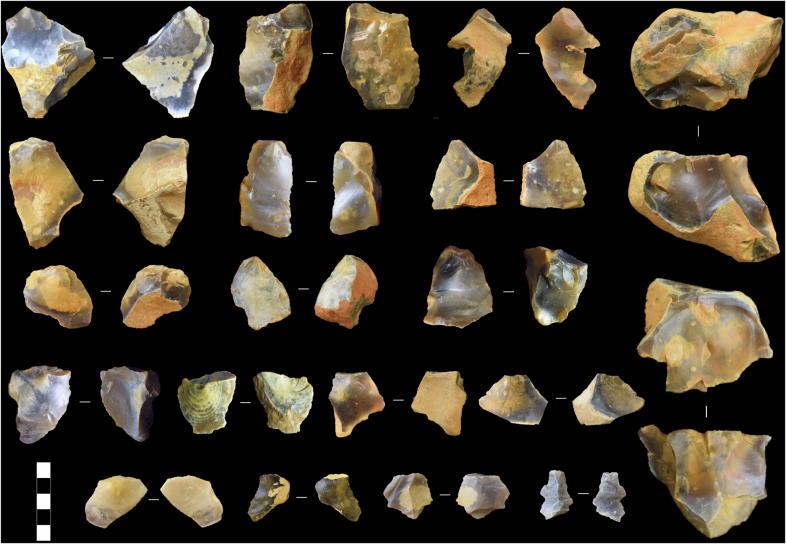
A larger selection of lithics from Trench Two and Three, relative to Fig. 3. Included are the two cores (right) and a series of flakes with varying degree of edge abrasion/rolling.

**Extended Data Fig. 3:**
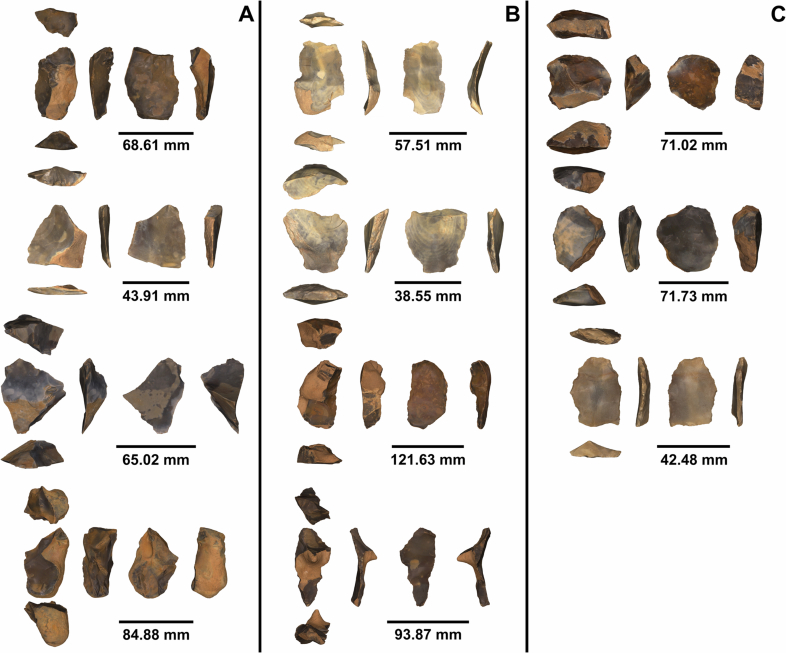
Flake artefacts and one core from Trench Two and Trench Three. A selection of flake artefacts and one core from Trench Two and Trench Three (**a** and **b**), along with surface found flakes from the gravels in the west of Old Park (**c**). These artefacts are specifically highlighted here as they have been 3D scanned and can be downloaded as individual .ply files in the supplementary information of this article. File names in the ply. files refer to this image. Note that the western-discovered artefacts were left on-site.

Here, three excavated trenches and a series of exposures and test trenches from a 1920s aggregate quarry contained within Old Park—known as Fordwich Pit^21,23–25^—are described. Preserved Quaternary sediment around the circumference of the quarry is demonstrated to be highly variable in depth, ranging from <2 m to >6 m (Fig. 2, Supplementary Fig. 2, Extended Data Fig. 1 and Supplementary Information). Along the northern to southwestern edges of the quarry, near the brow of the hill, three large excavated trenches and seven test trenches have been created, along with four exposures having been cleared (Supplementary Figs. 1 and 2). All but four of these have yielded artefacts at low frequencies (Fig. 3, Extended Data Figs. 2 and 3 and Supplementary Fig. 3). Comprising most of the remaining sediment at the edge of the quarry, these sand and gravel beds should be interpreted as part of the artefact-bearing braided river system described in earlier works^21,23–26^. Trench one represents a major extension to those previously described^25^, while trenches two and three are newly reported.

Trench one preserves two infrared-radiofluorescence (IR-RF) dated levels; upper gravels and sand lenses with dates clustering at approximately 372 ka, while the dates for the lower gravels cluster at about 542 ka (ref. ^25^). The younger age is interpreted to result from later reworking of the uppermost part of the gravel, while the older date may be reflective of the MIS 14 fluvial deposition of the gravel^25^ (but see below). More than 6 m^3^ of gravel was excavated to a depth of >2 m (Fig. 2). Inclined Palaeogene Lambeth Group sands are present at a depth of around 2.3 m (Fig. 2). Artefacts are technologically and stratigraphically in line with those described by Key et al.^25^; mostly flakes, recovered from both dated levels and characterized as taphonomically variable (rolled through to fresh) with very occasional, and cautiously interpreted (because of the fluvial location), signs of retouch (Supplementary Fig. 3). We are cautious about providing final sample sizes; the number of artefacts from trench one has grown since those described from the first fieldwork season^25^, but discovery frequency has decreased. We therefore re-emphasize our previous suggestion that some of the 251 artefacts reported in Key et al.^25^ are probably formed through past fluvial activity (Supplementary Fig. 4). Three sediment samples from one location in the lower MIS 14 level were collected for palaeoenvironmental analyses (Fig. 2 and Supplementary Tables 1 and 2). Although preservation was generally poor, phytolith evidence indicates a diverse assemblage, with Pooideae (temperate/C_3_) and Chloridoideae (arid/C_4_) grass leaf morphotypes and dicot types, although relative proportions require future assessment with better preserved assemblages.

Trench two proceeded to a maximum depth of 4.6 m in the southwest corner of the quarry immediately behind the southern Bridgland et al.^21^ exposure (Fig. 2). The fine sands, previously dated to MIS 12 via IR-RF^25^, and potentially representing redeposited Lambeth Group sands (Supplementary Information), were found to be wholly sterile of artefacts but retained the most diverse environmental evidence from all investigated layers/trenches: dicots and grasses including Pooideae, Chloridoideae and Panicoideae (temperate to tropical/C_4_). Samples were collected from the extreme lower limit of the sand (Fig. 2 and Supplementary Tables 1 and 2). The upper gravel, similarly previously dated through IR-RF to MIS 12 (ref. ^25^), and again probably redeposited during this glacial period, returned flake artefacts and two cores across its depth (~2 m) (Figs. 2 and 3 and Extended Data Fig. 2). Most were present at the extreme superior limit of the upper gravel (spit, 1.5–1.6 m), a few were found at 1.8–2.0 m, and one was discovered at its extreme lower limit (spit, 3.4–3.5 m). The flakes are morphologically and technologically diverse, and include forms consistent with the late-stages of producing heavily shaped handaxes, alongside those from biface ‘roughing-out’ or flake production (Fig. 3, Extended Data Fig. 3 and Supplementary 3D Models). Sediment varied, ranging from compacted medium flint gravels (3–7 cm) with occasional nodules >10 cm, through to pockets of loose fine gravel, lenses of fine-grained sand and sand layers; all consistent with a braided river system (compare ref. ^27^). Some of the flakes at the upper extreme of this cold-stage gravel, and in a vertically discrete layer immediately beneath the fine sand, are very fresh (that is, have sharp, non-rounded edges) (Fig. 3, Extended Data Figs. 2 and 3 and Supplementary 3D Models). Combined with the fluvial context, this suggests that the artefacts were deposited soon after being produced, quickly became covered and were exposed to little taphonomic alteration. Three phytolith samples were collected from three locations in the upper gravels (nine in total): within the first 10-cm spit of the gravel, at a depth of 340 cm immediately above a large sand layer/lens overlying the lower gravels, and from within this sand (Fig. 2). There was little difference between these samples; few grass morphotypes were observed and those that were identified belong to Pooideae, while dicots were more abundant in the two upper sampled layers, but were absent from the sand lens.

Together, the evidence could be interpreted as hominins knapping on an exposed, previously deposited gravel bank during MIS 12, before the superior fine sands being deposited in a low-energy environment during the same cold stage. The presence of artefacts within and at the base of the MIS 12 gravels (also refs. ^21,26^), combined with probably undisturbed artefacts on its superior surface, suggests two periods of hominin occupation at Old Park during MIS 12, or the lower part of the gravels retaining artefacts from MIS 13 in addition to a later re-occupation in MIS 12. Two occupation phases are supported by the palaeoecological data. The upper and lower extremes of these gravels indicate the presence of some temperate grasses and flowering plants, suggesting a temperate ecology when this upper sediment began and ceased to accumulate.

To better understand the age of the lower gravels in trench two and, in turn, artefacts seemingly previously discovered at this depth^21,23,24,26^, six new samples were dated via IR-RF (Fig. 2) and four samples were subject to palaeomagnetic analyses (Supplementary Information). During the collection of these IR-RF samples, one potential flake was identified from the lower gravels. One probable flake artefact was also excavated from the lower gravels of trench two at a depth of 3.6–3.7 m (Fig. 3). One of the lower two IR-RF samples was excluded because of insufficient coarse-grain K-feldspar. The remaining five samples identify the earliest sediment at Old Park, and probably some of the earliest artefact-bearing sediment in northern Europe (Table 1). The obtained IR-RF De (equivalent dose) values (mean out of three aliquots) range from 639 ± 38 grey (Gy) (sample 8) to 926 ± 15 Gy (sample 7). No clear stratigraphic associations exist between the results and the location of each sample. The 2 younger ages, associated with early MIS 15 and mid-MIS 16, link the deposition of the gravels to MIS 16 fluvial activity, and any artefacts within to MIS 17 (712–676 ka) or 16 (676–621 ka). These dates are consistent with previous fluvial incision and uplift estimates^21^. Three earlier dates cluster at 800 ka, suggesting a MIS 20 (814–790 ka) gravel deposition. These upper age estimates may reflect the use of the method at the upper end of its functional (temporal) range or an overestimation due to incompletely bleached IR-RF signals caused by rapid transportation and burial of sediment. Importantly, all dates precede those returned by Key et al.^25^. Alternating field (AF) and thermal (TH) palaeomagnetic demagnetization identified a normal magnetic polarity (Table 2). In combination with IR-RF ages, these data identify the Brunhes normal polarity, indicating a maximum deposition age of about 773 + 2 ka (ref. ^28^) (Supplementary Information).

**Table 1:** IR-RF ages returned from the six sediment samples collected from the lower gravels in trench two

Sample ID	Location in lower gravel	U (ppm)	Th (ppm)	K (%)	DR total (Gy ka^−1^)	De (Gy)	Age (ka)	Error (ka)	MIS association
3	Lower	1.00 ± 0.15	3.08 ± 0.14	0.26 ± 0.02	1.21 ± 0.15	780 ± 87	643	78	16
4	Lower	–	–	–	–	–	–	–	–
5	Centre	0.53 ± 0.09	1.62 ± 0.08	0.29 ± 0.02	1.15 ± 0.06	913 ± 23	795	43	20
6	Centre	0.62 ± 0.12	3.32 ± 0.10	0.30 ± 0.02	1.18 ± 0.06	921 ± 10	780	39	19
7	Upper	0.78 ± 0.12	1.92 ± 0.09	0.17 ± 0.01	1.12 ± 0.05	926 ± 15	826	40	21
8	Upper	0.51 ± 0.11	1.61 ± 0.08	0.13 ± 0.01	1.05 ± 0.08	639 ± 38	607	45	15

Two age clusters are present in the returned ages, with the oldest sediment sample aligning with MIS 21 and the youngest aligning with MIS 15. The variation in De among the samples probably links to varying degrees IR-RF signal bleaching. Such variance in the data probably reflects the use of the method at the upper end of its functional (temporal) range. The De data are mean values with its standard error. A table with the nuclide concentrations of the single samples is presented in Supplementary Information. For calculating the dose rate (DR) a water content of 20 ± 10% was used. The high error of 10% was chosen with respect to the uncertainties within the water content especially over the relevant Quaternary timescales.

**Table 2:** Palaeomagnetic directions recovered from AF and TH demagnetization of the lower gravel in trench two

Sample ID	Level	Type	Dec. (°)	Inc. (°)	MAD (°)	No. of steps	Steps (mT per °C)
K1.1	Lower gravel	AF	352	62	14	6	15, 20, 25, 30, 40, 50 mT
K1.2	Lower gravel	AF	356	62	11	6	15, 20, 25, 30, 40, 50 mT
K1.3	Lower gravel	AF	308	64	11	6	15, 20, 25, 30, 40, 50 mT
K1.5	Lower gravel	TH	NA	NA	NA	NA	NA

Sample ID, sample identification; Level, stratigraphic level of palaeomagnetic sample; Dec., declination of characteristic remanent magnetization (ChRM); Inc., inclination of ChRM; MAD, maximum angular deviation; No. of steps, number of steps used for calculating ChRM; Steps, demagnetization step used to calculate ChRM. The thermal sample indicated a normal polarity at lower temperatures but disintegrated at higher temperatures and did not give a useable result (NA).

**Extended Data Fig. 4:**
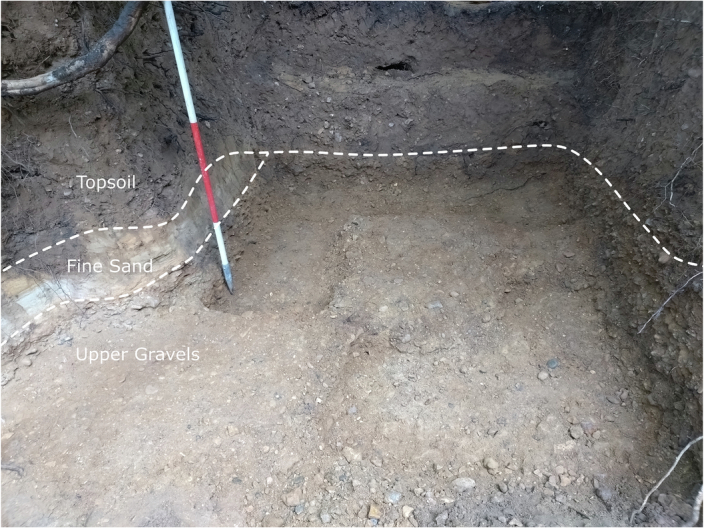
Schematic depiction of Trench Three. Note the fine sand on the left (southern edge) but it’s absence on the right (northern edge).

Trench three identified the most northern and eastern limits of the remaining fine sands of the site (Supplementary Figs. 1 and 2 and Extended Data Fig. 4). Beneath 1.1 m of top soil, up to 30 cm of sand was present at the southern edge of the trench, before it rapidly decreased in depth and ceased by the northern limit of the trench. Immediately beneath the fine sand flake, artefacts were discovered in the upper gravels (1.3–1.7 m), including one biface-thinning flake (Fig. 3, Extended Data Fig. 4 and Supplementary 3D Models). IR-RF and phytolith sampling was not undertaken in trench three.

Our interpretation of the wider quarry is that the lower MIS 16 gravels potentially covered most of the site, while the younger MIS 12 gravels and sands were restricted to more westerly portions of the pit, potentially due to the presence of a Stour tributary running parallel to the site and about 30 m from trenches two and three (as previously reported^25^) (Fig. 2 and Supplementary Fig. 2). This accords with the marked east-to-west increase in gravel depth^24,25,29^ and the lack of any MIS 12 sediments in the more easterly trench one. In turn, and given the greater number of artefacts discovered in the western edge of the quarry near the brow of the hill^24,29^, the upper gravels were probably responsible for a substantial proportion of the artefacts discovered in the 1920s. Further, the MIS 16 age of the lower gravels in trench two, and the near-identical vertical alignment of the lower gravels in trench two and the gravels in trench one (Fig. 2), raises the possibility that the MIS 14-age of the lower gravels in trench one could have resulted from reworking, as with the upper gravels in trench one^25^, after having originally been deposited during MIS 16. In turn, the artefacts in the upper and lower gravels of trench one could be derived from MIS 16, with the presence of flakes through the trench one sequence subsequently supporting the presence of artefacts throughout the lower gravels in trench two. Thus, there are probably two, but potentially up to four, periods of occupation at Old Park (Figs. 1 and 2). An earlier (probably MIS 17–16) hominin presence, followed by a later cold-stage occupation evidenced by the upper levels of the MIS 12 gravels in trench two. A third, early MIS 12 or MIS 13 occupation could also be evidenced by the artefacts in the lower levels of the MIS 12 gravels. Finally, hominin presence could also be evidenced in the MIS 14 gravels, but equally, recovered artefacts could reflect the earlier occupation of the site.

**Fig. 4:**
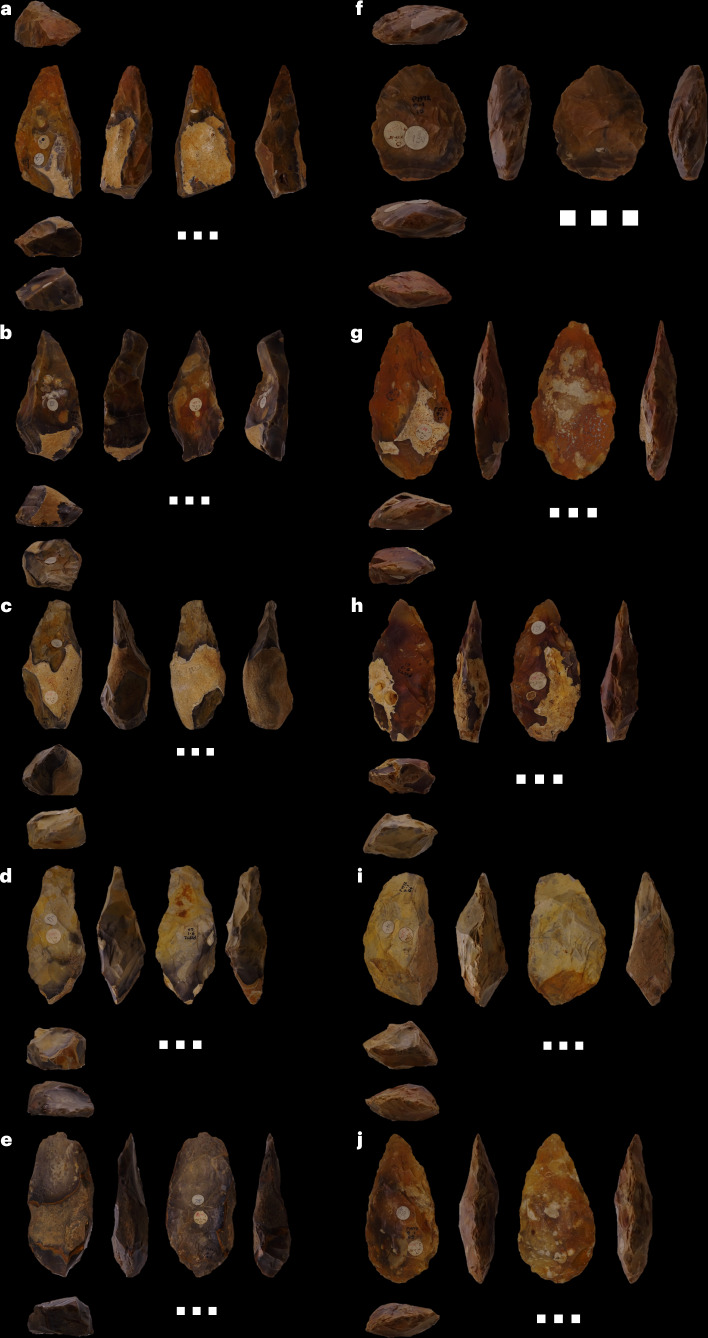
Ten handaxes recovered from Fordwich Pit as part of the 1920s aggregate quarrying. **a**–**j**, Note the heavy flaking and shaping investment observed in **f** to **j** (right) relative to **a** to **e** (left). Tranchet flake removals, strongly associated with MIS 13 at other British Acheulean sites, can be seen on images **f**, **h** and **j**. Trihedral and quadrihedral forms can be seen in images **a**, **b** and **d**. Scale bars, 5 cm.

**Extended Data Fig. 5:**
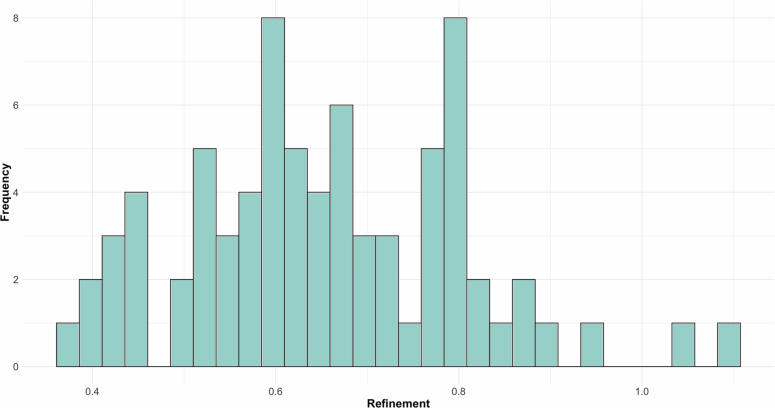
The relative thinness (refinement) of a sub-set of 76 handaxes from the historically collected assemblage from Fordwich Pit. Note the bimodal distribution in support of the presence of artefacts produced by two distinct populations.

**Extended Data Fig. 6:**
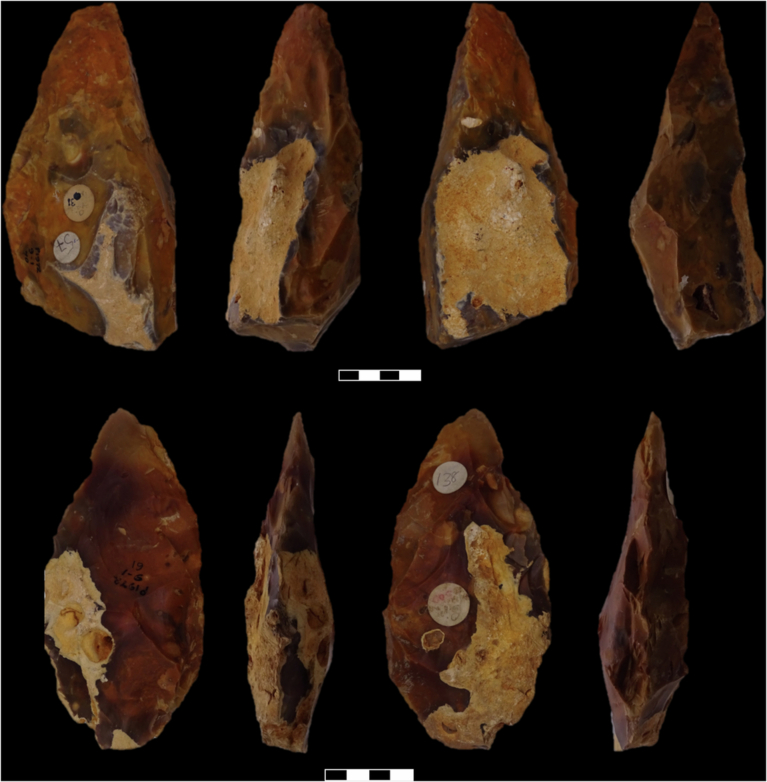
Handaxes from Fordwich Pit. Exemplar handaxes from Fordwich Pit displaying a tranchet flake removal (bottom), which we infer to likely be derived from the MIS 12 sediment in our excavated trenches, and a trihedral pick-like form that is atypical for the British Acheulean during MIS 15, 13 and 11, and which we infer to be from our excavated MIS 16 dated sediment (top).

To contextualize the historically collected handaxes in light of these new data, we recorded technological and morphological information from this existing assemblage. Bimodal distributions were identified in multiple regards, suggesting that the Fordwich Pit handaxes derive from two populations (Fig. 4, Supplementary Information and Extended Data Fig. 5). Elongated and often thick forms with relatively low scar counts and tip-targeted removals, which includes trihedral and quadrihedral specimens atypical for Britain during MIS 15 to MIS 11, were identified, supporting a MIS 17/16 Acheulean presence (Fig. 4 and Extended Data Fig. 6). Equally, a sizeable sample of ovate specimens with clear use of soft hammer flaking, including tranchet removals, was identified; a technological marker of the British MIS 13 record, especially from Boxgrove^30^, thus aligning with the aforementioned second, later MIS 12 occupation at Old Park (Fig. 4 and Extended Data Fig. 6).

## Discussion

Old Park is one of the earliest archaeological contexts of the UK, and arguably the earliest with accessible, excavatable artefact-bearing sediments. Hominin presence is suggested from MIS 17–16, with a later re-occupation in MIS 12 and potentially MIS 13. During the later ~437-ka period there is convincing evidence of occupation by Acheulean hominins at 51° latitude during the Anglian glacial stage. Substantial, undisturbed Lower Palaeolithic artefact-bearing sediment of similar ordnance datum, and therefore probably age, to those dated here, remain across the Old Park SSSI.

The importance of Old Park is further emphasized by the >330 handaxes recovered from the Fordwich Pit gravels in the 1920s^23,24,31^. The age of these bifaces is important to understanding the emergence of Acheulean technology in northern Europe^8,29,32,33^. For decades, the large number of ‘rough’, lightly worked forms in the assemblage, along with their high-terrace origin, resulted in pre-Anglian age inferences^21,25,34^. Previous work in trench one suggested that they could have been derived from MIS 14 gravels^25^, but the stratigraphy of trench two more closely matches Smith’s description^24^ of the interstratified gravels and sands from which the handaxes were recovered (Fig. 2). Its location on the western edge of the quarry further supports its close association with the original discoveries^24,25,29^. Our discovery of biface-thinning flakes in the upper gravels of trenches two and three strongly supports the presence of handaxes in these locations (an inference supported by others (for example, N. Ashton, personal communication; Supplementary Information). Some of the Old Park (Fordwich Pit) handaxes are, therefore, probably derived from MIS 13–12.

Few contemporary records of the handaxe’s recovery exist, with a flood in 1953 destroying any provenance information that did exist^29,35^. The biface assemblage has, however, always been characterized as being unusually diverse. ‘Rough’, lightly worked and irregularly formed handaxes, often elongated, are present alongside more heavily flaked and intensively shaped forms of diverse morphologies^31^ (Fig. 4). Roe^29^ noted “the Fordwich implements lack all refinement of technique with the exception only of a couple of refined ovates, quite out of character…”. Ashmore^31^ (page 102) stated “The crudeness of manufacture, irregularity and narrowness… has always been emphasised… However, it is well worth pointing out that there are also distinct types… which are well flaked, often finished with a soft hammer technique and which certainly cannot be thought of as crude in manufacture…”. We propose that from this bimodal distribution, the rougher forms may derive from the older, lower gravels, while the more heavily worked forms could have been discovered in the superior, younger gravels (see Moncel et al. and Davis et al.^12,36^ for similar inference). Certainly, the rough forms are consistent with some of the ‘crude’ MIS 17/16 bifaces known from la Noira, France, while the trihedral and quadrihedral specimens are arguably akin to the pick-like forms seen at the late early Pleistocene site of La Boella (Spain)^3,8,33,37^(Fig. 4 and Supplementary Information). Conversely, the heavily shaped ovates, some of which show tranchet flaking, can be considered technologically and morphologically typical of later MIS 13–12 handaxes found widely across Britain^12,30,38^ (Fig. 4 and Extended Data Fig. 6). Several newly discovered biface-thinning flakes have multiple dorsal extractions, further supporting the MIS 12 presence of highly shaped handaxes. Through both periods, Old Park showed environmental conditions that were probably suitable for Acheulean populations (Fig. 1). Potentially, therefore, Old Park simultaneously retains the earliest evidence for Acheulean artefacts in northern Europe, with handaxes derived from MIS 16–17, along with more technologically advanced forms produced about 200,000 years later.

Combined with artefactual evidence from Moulin Quignon^8,10^, environmental data from Valle Giumentina^39^, Happisburgh and Pakefield^2,7^, and elsewhere^40^, as well as recent palaeoenvironmental modelling^41,42^, Old Park supports growing evidence for early-to-mid-Pleistocene hominins being able to occupy European high latitudes during glacial periods and/or cool-to-cold climates. Old Park hominins could, therefore, have had the cultural and technological attributes necessary to survive in these cooler climates and ecologies^43^. Although taphonomic issues need to be taken into account, our palaeoecological data suggest that a mix of grassland subfamilies, as well as flowering plants, were present during the MIS 12 occupation. The most harmonious interpretation of evidence from Old Park may, therefore, be an occupation during an MIS 12 interstadial, but hominin presence during other MIS 12 periods cannot be ruled out. Occupation was more likely when glaciers were not at their southern limits around 65 km north of Old Park^44^, but we do not have the resolution to rule this out entirely. Hominin populations supported by grasses and grassland fauna, alongside forest and woodland, are noted elsewhere in Britain during MIS 13 and early MIS 12, including at Boxgrove and High Lodge^40,45^. Old Park is, however, notable for lacking evidence of woodland and forest ecologies, hinting at a cooler, dryer continental climate and grassland ecology, which accords with the late MIS 12 age, potentially summer-only occupation and even ice-proximal conditions (Fig. 1), but poor phytolith and faunal preservation could be obscuring this signal.

Following other work^8,15^, Old Park suggests against inferring that artefacts discovered in northern European glacial-stage Quaternary gravels were produced in the preceding warm stage, with both glacial and interglacial occupation appearing possible. Contrary to our previous suggestion^25^, the artefacts present in trench one do not, therefore, necessarily derive from MIS 15, but were produced during either MIS 15 or 14, or were reworked from the older sediments evidenced to the west of the trench. Similarly, artefacts from the trench two lower gravels may derive from MIS 17 or 16. In a 1932 letter contemporary to the earliest Old Park discoveries, Willock states “the bulk of the implements are in fresh condition” (cited in ref. ^29^: page 14), while Ashmore^31^ stresses one-quarter of the handaxes of the site to be “fresh”. Taphonomic assessments consistent with our own observations (Supplementary Information). If sharp, taphonomically unaltered implements at Old Park can be associated with glacial occupation—as they can in the upper gravels of trench two—then a substantial proportion of the Acheulean material may be linked to cold-to-cool climates, representing a rare opportunity to investigate mid-Pleistocene hominin behaviour in such conditions.

Taken together, it is likely that the banks of the ancient Stour river were repeatedly occupied by hominin populations during the mid-Pleistocene. Old Park probably preserves rare evidence of hominin presence in northern Europe from MIS 17 to MIS 16 (712–621 ka), during MIS 13–12—potentially on two occasions, but at least once—and possibly also during MIS 15–14 (563–533 ka), depending on the interpretation of trench one. The MIS 12 sediment is important for its evidence of high-latitude Anglian-stage occupation by mid-Pleistocene Acheulean hominins. Phytolith data suggest a mixed grassland environment, potentially indicating occupation during an interstadial. The MIS 17–16 dated gravels are important for their association with the substantial, but technologically and morphologically varied, handaxe assemblage recovered in the 1920s. The rougher, more irregularly flaked handaxes in this assemblage potentially represent the earliest known handaxes from northern Europe, while the more heavily flaked forms may reveal a re-occupation by Acheulean populations about 200,000 years later.

## Methods

### Excavation

Sediments at the Old Park quarry site were mostly deposited through fluvial processes. As such, the three-dimensional plotting of recovered artefacts was not undertaken and excavations proceeded by hand through the removal of 10-cm spits. At its superior level, trench one measured 2 × 3 m^2^, before increasing to approximately 3 × 3 m^2^ at its lowest depth, owing to the sloped bank of the quarry at its lowest depth (Fig. 2). Trench two measured 3 × 4 m^2^ at its superior point, before decreasing to 1 × 2 m^2^, and eventually 1 × 1 m^2^, due to stepping (Fig. 2). Trench three measured 2 × 3 m^2^ (Extended Data Fig. 4). All sediment was sieved through 6-mm screens. Any recovered artefacts were assigned with their respective spit. Where possible, flakes were recovered in situ and bagged immediately. Additionally, seven test trenches and four exposures were machine dug around the perimeter of the quarry. Artefacts discovered in situ are interpreted as probably being derived from the MIS stage associated with the relevant sediment or, in the case of glacial-stage gravels, potentially also from the preceding warm stage, unless otherwise specified. Elevation was recorded using a Leica Geosystems 1200 Differential GPS system. Results were processed using Leica Infinity software and reported with respect to the Ordnance Survey OSGM15 geoid model. Additional information concerning the excavation and recovery of artefacts can be found in Supplementary Information.

### Lithic artefacts

We report technological information on 18 lithic artefacts recovered from the upper gravel of trench two. We use this level and trench to provide exemplar artefacts from Old Park as they have not previously been described. We do not describe all artefacts recovered to date to avoid misinterpretation and subsequent misreporting in the literature as work is ongoing and assemblage proportions could change in the short term (but see Supplementary Figs. 2 and 3 and Extended Data Figs. 2 and 3 for additional examples). A comprehensive review of all lithic material will be produced once the present course of fieldwork is complete. We are confident in the assignment of these artefacts as intentionally knapped objects, but re-acknowledge^25^ the complications created by their fluvial deposition and note that some recovered lithic objects not presented here may lean towards a natural origin (Supplementary Fig. 4). This trench two assemblage is most often characterized by simple—and technologically undiagnostic—flaking strategies, seemingly mostly using locally available flint from the ancient gravels of the Stour, although banded flint known to be eroding from the Kent Downs several miles away is also identified (for example, Fig. 3a; Supplementary 3D Models B1 and B2). Flakes show proximal and orthogonal reduction sequences and up to seven dorsal scars (Supplementary Table 1). Pronounced cones/bulbs of percussion and relatively thick platforms could be interpreted as being characteristic of hard hammer percussion and internal knapping motions. One flake from this subsample is consistent with the late-stages of producing highly shaped handaxes through the removal of ‘thinning’ flakes, as it has a small platform, diffused bulb, four dorsal scars and is thin, curved and elongated in form (Fig. 3). The two cores are consistent with the flake assemblage, being interpreted as river-sourced flint and having low scar counts. One core shows shattering caused by internal fractures. The lithics have low levels of abrasion/chipping, indicating little-to-no reworking from their depositional context. Flakes identified during wider surveys of the Old Park area (Supplementary Fig. 1) are consistent with the excavated artefacts and exhibit similar surface patinas, but some are heavily rolled.

### IR-RF dating

**Extended Data Fig. 7:**
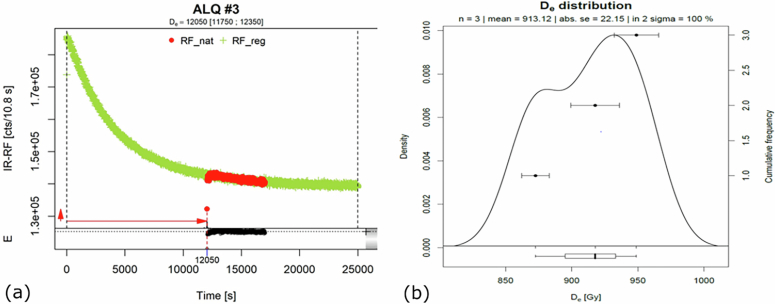
Natural and regenerated IR-RF signal obtained on sample 8 (2A). The IR-RF intensity is defined by the probability of electron-trapping per time interval. RF data analysis was conducted using the R- luminescence/ IR-RF package (version v. 3.5.1; Kreutzer et al. 2012) using the sliding technique (horizontal and vertical slide). Final IR-RF De values are based on the mean value of all measured aliquots (A). For IR-RF De-measurements, 5 mm sized aliquots were used. IR-RF DE- distribution obtained from sample 3 (B).

To further constrain the chronology of the lowest gravel and sand unit in trench two, which probably contains both reworked and in situ artefacts^21,23–26^, an additional six samples were taken for IR-RF dating^46,47^ from the new excavations at the southwestern edge of the quarry (Extended Data Figs. 7 and 10 and Table 1). By dating the fluvial deposits around the artefacts, minimum ages can be delivered. A detailed description of the methodological approach can be found in Supplementary Information.

### Palaeomagnetic analyses

**Extended Data Fig. 8:**
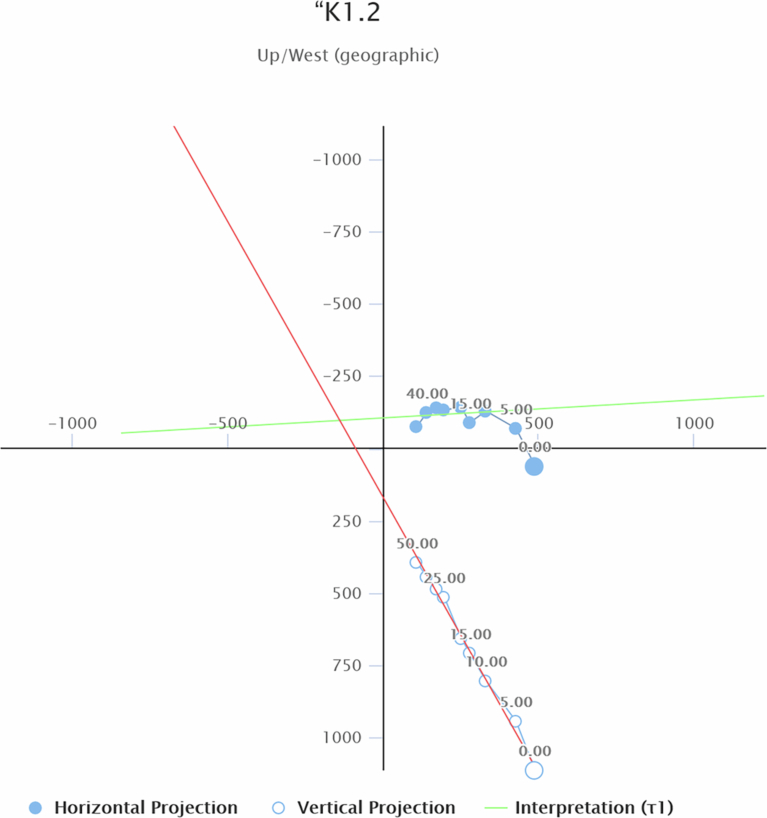
An orthographic projection (Zijderveld diagram) showing the AF demagnetization directions for specimen K1.2. Closed circles show the declination of the specimen demagnetization and open circles show the inclination of the specimen demagnetization. The green and red lines show the best fit to the declination and inclination, respectively. The distance from the origin represents the magnetic moment. Demagnetization reveals a high-coercivity component between (15-50 mT) showing a direction of (356, 62) indicating normal polarity, suggesting the specimen’s deposition and magnetization post-dates the Brunhes-Matuyama reversal 773 ± 2 ka.

**Extended Data Fig. 9:**
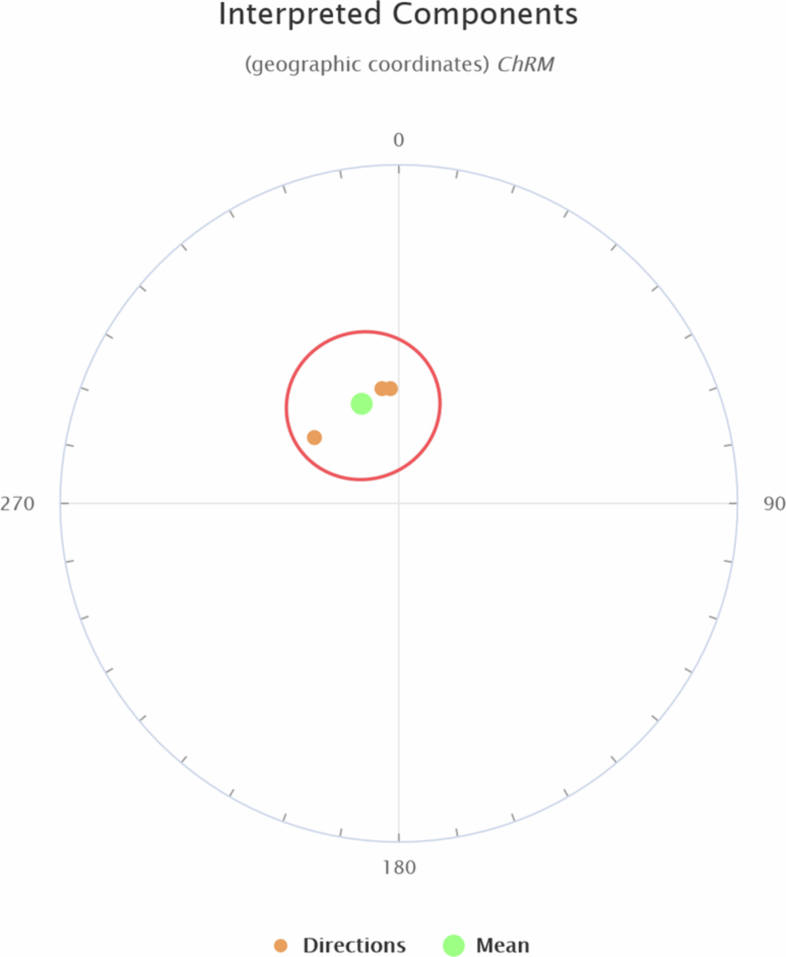
An equal area, lower hemisphere stereographic projection showing the recovered high coercivity characteristic remanent magnetization (ChRM) directions for specimens K1.1, K1.2 and K1.3 (orange circles). The closed green circle is the mean direction and the red circle is the α95 uncertainty (that is, there is a 95% probability that the direction lies within the red circle). Closed circles indicate that the directions represent a normal polarity (positive inclination), again supporting deposition and magnetization following the Brunhes-Matuyama reversal c. 790 ka.

**Extended Data Fig. 10:**
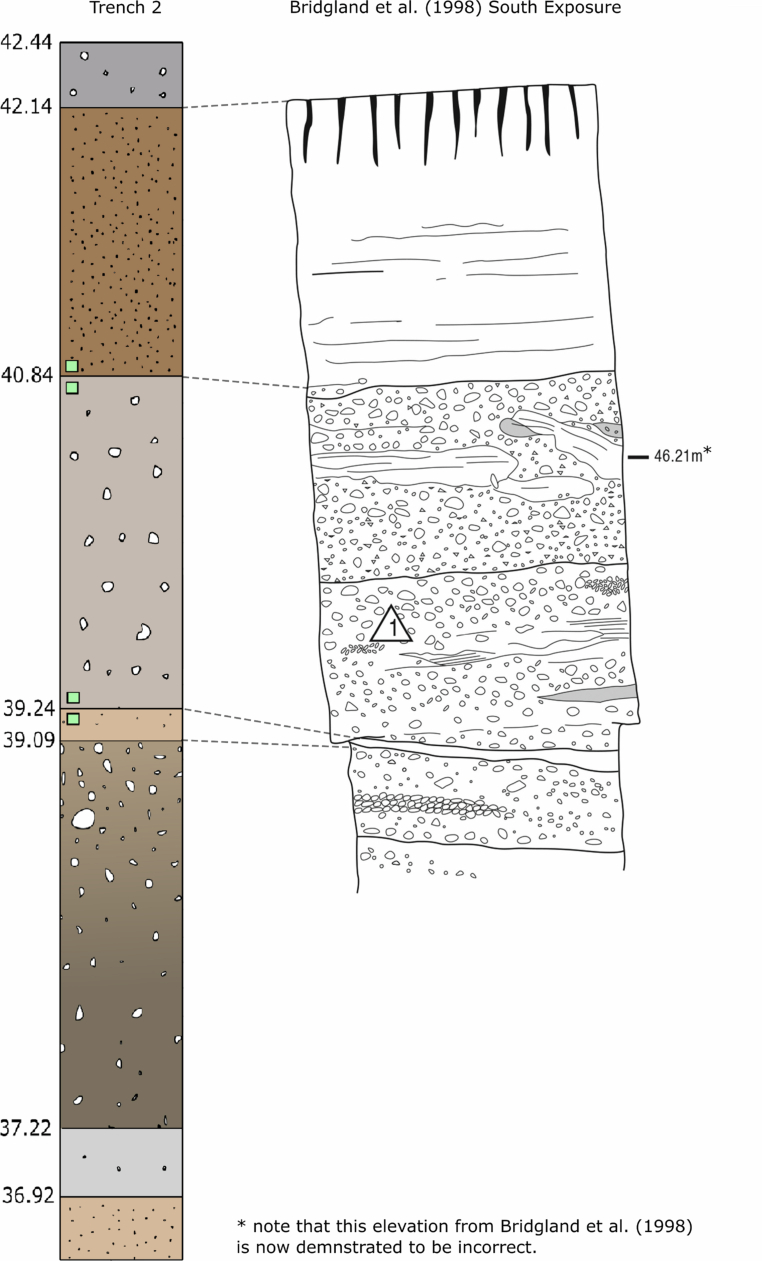
Elevation data from Trench 2. Alignment of elevation data from Trench 2 and the southern exposure located two meters to the north, as published by Bridgland et al. (1998).

Identifying the palaeomagnetic polarity of the lower gravels helps to constrain its chronology and the date of deposition of the artefact (Extended Data Figs. 8 and 9). The lower gravel is estimated to have been deposited around the Brunhes–Matuyama reversal dated at 773 + 2 ka (ref. ^28^). A normal magnetic polarity will indicate an age younger than this reversal, while a reversed magnetic polarity will indicate an age older than this reversal. A detailed description of the palaeomagnetic methods can be found in Supplementary Information.

### Palaeoenvironmental (phytolith) data

Phytoliths were extracted from 3 samples in trench one and 12 samples in trench two (methodology in Supplementary Information). These samples are from fluvial or near-fluvial contexts, rather than anthropically created ones, and thus water-transportation and dissolution should be accounted for. Although a relatively heavy microfossil, phytoliths can and are moved by water, and will be selectively sorted by such actions^48^. The phytoliths of interest in this study are, however, short cells of grass that are the same size and weight, and are unlikely to have been selectively sorted. Comparisons with more ornamented types, such as hairs, or heavier forms, such as bulliforms, are not suitable for study. Dissolution should also be considered—although typically resistant to chemical change over prolonged periods (as in the case of MIS-length studies), phytoliths have the potential to have sites of damage^49,50^. However, Cabanes and Shahack-Gross^51^ suggest short cells, such as those seen here, to be relatively stable in shape (that is, less affected by dissolution and other postdeposition taphonomic factors), and can be used for tentative reconstructions of the palaeoenvironment.

Phytoliths also typically represent the local environment. Madella and Lancelotti^48^ note that ‘heavy’ microfossils phytoliths do not generally travel far beyond the place of plant necrolysis, although where there is heavy runoff or fluvial action this can be altered. As this is a fluvial environment, we have to consider that we may not be looking at a directed local environment but one that represents the river valley region at a broader brush-stroke picture. This, however, has the positive side effect of providing a wider insight into the hominin palaeoenvironment rather than just this sediment column.

With this considered, we cautiously use the palaeoenvironmental data to explore the broader environmental setting of each MIS stage, localized to this fluvial region. That said, preservation/numbers of phytoliths found was poor (Supplementary Table 1), as expected in a non-anthropic sediment^48,51,52^. A qualified presence-only analysis was therefore carried out rather than full quantification to reduce the chance of over interpretation. Short cells of grasses were most abundant (relatively). As Cabanes and Shahack-Gross^51^ note, short cells are relatively stable in shape, and as a result it is cautiously argued that the samples showed that throughout the three dated cold-stage gravels grasses were present, with some small differences observed and outlined in Supplementary Table 2.

A range of grass subfamilies including Pooideae, Chloridoideae and Panicoideae are seen in the deep samples dated to both MIS 14 (trench one) and MIS 12 (trench two). The uppermost sample of trench two has the broadest range of morphotypes (Supplementary Table 3), and it should be noted that the best preservation was seen in this sample (less degradation and damage). This could imply that the deep samples are reduced in morphotype range not due to environmental differences but due to taphonomic processes such as weathering, chemical damage or leaching^48,52^, although testing of exactly how reworking, dissolution or movement in the sediment column may have worked is needed. It is worth noting that the lower samples produced ‘dirty slides’ from excess microsilica fragments, which means that the weights provided in Supplementary Table 2 are potentially misleading, particularly given the small number of phytoliths actually seen. Despite these caveats, there are identifiable plant groups and similarities between trenches one and two that highlight the need for further sampling.

### Reporting summary

Further information on research design is available in the Nature Portfolio Reporting Summary linked to this article.

## Supplementary information

Supplementary Information Supplementary text, Tables 1–3 and Figs. 1–7.

Reporting Summary

Supplementary Data 3D models of artefacts.

## Data Availability

All data are available in the main text or Supplementary Information, or via cited open access references. The artefacts are currently housed at the University of Cambridge for analysis (access via the corresponding author) but will be accessioned with a yet-to-be-finalized museum in the long term. The geochronological and palaeoenvironmental analyses are destructive but repeat sampling is possible with relevant permissions. All 3D models are available as Supplementary Data.

## Code availability

No code was created for the manuscript. Any code used is available via cited open access references.

## References

[1] Roberts, P. & Stewart, B. A. Defining the ‘generalist specialist’ niche for Pleistocene *Homo sapien*s. *Nat. Hum. Behav.* **2**, 542–550 (2018). 31209320 10.1038/s41562-018-0394-4

[2] Parfitt, S. A. et al. Early Pleistocene human occupation at the edge of the boreal zone in northwest Europe. *Nature* **466**, 229–233 (2010). 20613840 10.1038/nature09117

[3] Moncel, M.-H. et al. Early evidence of Acheulean settlement in Northwestern Europe—La Noira site, a 700,000 year-old occupation in the centre of France. *PLoS ONE* **8**, e75529 (2013). 24278105 10.1371/journal.pone.0075529 PMC3835824

[4] Key, A. & Ashton, N. Hominins likely occupied northern Europe before one million years ago. *Evol. Anthropol.* **32**, 10–25 (2023). 36383204 10.1002/evan.21966

[5] Bermúdez de Castro, J. M. & Martinón-Torres, M. A new model for the evolution of the human Pleistocene populations of Europe. *Quat. Int.* **295**, 102–112 (2013).

[6] Galway-Witham, J., Cole, J. & Stringer, C. Aspects of human physical and behavioural evolution during the last 1 million years. *J. Quat. Sci.* **34**, 355–378 (2019).

[7] Parfitt, S. A. et al. The earliest record of human activity in northern Europe. *Nature* **438**, 1008–1012 (2005). 16355223 10.1038/nature04227

[8] Moncel, M.-H. et al. Were hominins specifically adapted to north-western European territories between 700 and 600 ka? New insight into the Acheulean site of Moulin Quignon (France, Somme Valley). *Front. Earth Sci.* **10**, 882110 (2022).

[9] Antoine, P. et al. Dating the earliest human occupation of Western Europe: new evidence from the fluvial terrace system of the Somme basin (Northern France). *Quat. Int.* **370**, 77–99 (2015).

[10] Antoine, P. et al. The earliest evidence of Acheulian occupation in Northwest Europe and the rediscovery of the Moulin Quignon site, Somme Valley, France. *Sci. Rep.* **9**, 13091 (2019). 31511611 10.1038/s41598-019-49400-w PMC6739401

[11] Duval, M. et al. A multi-technique dating study of two Lower Palaeolithic sites from the Cher Valley (Middle Loire Catchment, France): Lunery-la Terre-des-Sablons and Brinay-la Noira. *Quat. Int.* **556**, 79–95 (2020).

[12] Davis, R., Ashton, N., Hatch, M., Hoare, P. G. & Lewis, S. G. Palaeolithic archaeology of the Bytham River: human occupation of Britain during the early Middle Pleistocene and its European context. *J. Quat. Sci.* **36**, 526–546 (2021).

[13] Lewis, S. G. et al. A revised terrace stratigraphy and chronology for the early Middle Pleistocene Bytham River in the Breckland of East Anglia, UK. *Quat. Sci. Rev.* **269**, 107113 (2021).

[14] Mosquera, M., Ollé, A. & Rodríguez, X. P. From Atapuerca to Europe: tracing the earliest peopling of Europe. *Quat. Int.* **295**, 130–137 (2013).

[15] Key, A. Regional extinction(s) but continental persistence in European Acheulean culture. *Camb. Prism. Extinct.* **2**, e12 (2024). 40078812 10.1017/ext.2024.13 PMC11895719

[16] Gowlett, J. A. J. The early settlement of northern Europe: fire history in the context of climate change and the social brain. *CR Palevol.* **5**, 299–310 (2006).

[17] Van Kolfschoten, T., Parfitt, S. A., Serangeli, J. & Bello, S. M. Lower Paleolithic bone tools from the ‘Spear Horizon’ at Schöningen (Germany). *J. Hum. Evol.* **89**, 226–263 (2015). 26653208 10.1016/j.jhevol.2015.09.012

[18] Ollé, A. et al. The Acheulean from Atapuerca: three steps forward, one step back. *Quat. Int.* **411**, 316–328 (2016).

[19] Ashton, N. & Davis, R. Cultural mosaics, social structures, and identity: the Acheulean threshold in Europe. *J. Hum. Evol.* **156**, 103011 (2021). 34102521 10.1016/j.jhevol.2021.103011

[20] Lycett, S. J. & Gowlett, J. A. J. On questions surrounding the Acheulean ‘tradition’. *World Archaeol.* **40**, 295–315 (2008).

[21] Bridgland, D. R. et al. in *The Quaternary of Kent and Sussex* (eds Murton, J. B. et al.) (Quaternary Research Association, 1998).

[22] Chauhan, P. R. et al. Fluvia deposits as an archive of early human activity: progress during the 20 years of the Fluvial Archives Group. *Quat. Sci. Rev.* **166**, 114–149 (2017).

[23] Dewey, H. & Smith, R. A. Flints from the Sturry gravels, Kent. *Archaeologia* **74**, 117–136 (1924).

[24] Smith, R. A. Implements from high-level gravels near Canterbury. *Proc. Prehist. Soc. East Anglia* **7**, 165–170 (1933).

[25] Key, A. et al. On the earliest Acheulean in Britain: first dates and in-situ artefacts from the MIS 15 site of Fordwich (Kent, UK). *R. Soc. Open Sci.* **9**, 211904 (2022). 35754990 10.1098/rsos.211904 PMC9214292

[26] Briant, R. M. et al. Quaternary rivers, tufas and mires of Southern England: description of Geological Conservation Review sites. *Proc. Geol. Assoc.* **136**, 101084 (2025).

[27] Bridgland, D. R. *Quaternary of the Thames* (Springer, 1994).

[28] Channell, J. E. T., Singer, B. S. & Jicha, B. R. Timing of Quaternary geomagnetic reversals and excursions in volcanic and sedimentary archives. *Quat. Sci. Rev.* **228**, 106114 (2020).

[29] Roe, D. A. British Lower and Middle Palaeolithic handaxe groups. *Proc. Prehist. Soc.* **34**, 1–82 (1968).

[30] Roberts, M. B. et al. Boxgrove, West Sussex: rescue excavations of a Lower Palaeolithic landsurface (Boxgrove Project B, 1989–91). *Proc. Prehist. Soc.* **63**, 303–358 (1997).

[31] Ashmore, A. M. The typology and age of the Fordwich handaxes. *Cantiana* **96**, 83–117 (1980).

[32] Moncel, M.-H. & Ashton, N. in *The Emergence of the Acheulean in East Africa and Beyond* (eds Gallotti, R. & Mussi, M.) 215–235 (Springer, 2018).

[33] Ollé, A. et al. The earliest European Acheulean: new insights into the large shaped tools from the late Early Pleistocene site of Barranc de la Boella (Tarragona, Spain). *Front. Earth Sci.* **11**, 1188663 (2023).

[34] Wenban-Smith, F. & Cuming, P. *The Stour Basin Palaeolithic Project* (Archaeology Data Service, 2018).

[35] Knowles, P. G., Wickstead, H. & White, M. J. Tom Armstrong Bowes, Herne Bay Museum and the Lower Palaeolithic of the Kentish Stour. *Antiq. J.* **104**, 1–18 (2024).

[36] Moncel, M.-H. et al. The Early Acheulian of north-western Europe. *J. Anthropol. Archaeol.* **40**, 302–331 (2015).

[37] Mosquera, M. et al. The early Acheulean technology of Barranc de la Boella (Catalonia, Spain). *Quat. Int.* **393**, 95–111 (2016).

[38] Emery, K. *A Re-examination of Variability in Handaxe Form in the British Palaeolithic*. PhD thesis, Univ. College London (2010).

[39] Villa, V. et al. Environmental changes and human occupations between MIS 15 and MIS 14 in Central Italy: archaeological levels AO1-20, 24 and LBr of Valle Giumentina (c. 570–530 ka). *Archaeol. Anthropol. Sci.* **16**, 33 (2024).

[40] Candy, I., Schreve, D. & White, T. S. MIS 13–12 in Britain and the North Atlantic: understanding the palaeoclimatic context of the earliest Acheulean. *J. Quat. Sci.* **30**, 593–609 (2015).

[41] Rodríguez, J., Willmes, C., Sommer, C. & Mateos, A. Sustainable human population density in Western Europe between 560.000 and 360.000 years ago. *Sci. Rep.* **12**, 6907 (2022). 35484382 10.1038/s41598-022-10642-w PMC9051054

[42] Leonardi, M., Lycett, S. J., Manica, A. & Key, A. The Acheulean niche: climate and ecology predict handaxe production in Europe. Preprint at *bioRxiv*10.1101/2024.07.19.604259 (2024).

[43] Hosfield, R. Walking in a winter wonderland? Strategies for Early and Middle Pleistocene survival in midlatitude Europe. *Curr. Anthropol.* **57**, 653–682 (2016).

[44] Allen, P. et al. Mid-Late Quaternary fluvial archives near the margin of the MIS 12 glaciation in southern East Anglia, UK: amalgamation of multi-disciplinary and citizen-science data sources. *Quaternary* **5**, 37 (2022).

[45] Ashton, N. & Lewis, S. G. The environmental contexts of early human occupation of northwest Europe: the British Lower Palaeolithic record. *Quat. Int.* **271**, 50–64 (2012).

[46] Lauer, T. et al. Infrared radiofluorescence (IR-RF) dating of middle pleistocene fluvial archives of the Heidelberg Basin (Southwest Germany). *Geochronometria* **38**, 23–33 (2011).

[47] Murari, M. K. et al. Infrared radiofluorescence (IR-RF) dating: a review. *Quat. Geol.* **64**, 101155 (2021).

[48] Madella, M. & Lancelotti, C. Taphonomy and phytoliths: a user manual. *Quat. Int.* **275**, 76–83 (2012).

[49] Albert, R. M., Weiner, S., Bar-Yosef, O. & Meignen, L. Phytoliths in the Middle Palaeolithic deposits of Kebara Cave, Mt Carmel, Israel: study of the plant materials used for fuel and other purposes. *J. Archaeol. Sci.* **27**, 931–947 (2000).

[50] Esteban, I. et al. Palaeoenvironments and plant availability during MIS 6 to MIS 3 on the edge of the Palaeo-Agulhas Plain (south coast, South Africa) as indicated by phytolith analysis at Pinnacle Point. *Quat. Sci. Rev.* **235**, 105667 (2020).

[51] Cabanes, D. & Shahack-Gross, R. Understanding fossil phytolith preservation: the role of partial dissolution in paleoecology and archaeology. *PLoS ONE* **10**, e0125532 (2015). 25993338 10.1371/journal.pone.0125532 PMC4439089

[52] Cabanes, D., Weiner, S. & Shahack-Gross, R. Stability of phytoliths in the archaeological record: a dissolution study of modern and fossil phytoliths. *J. Archaeol. Sci.* **38**, 2480–2490 (2011).

