# Supplementary material for: Hominin glacial-stage occupation 712,000 to 424,000 years ago at Fordwich Pit, Old Park (Canterbury, UK)
Source: Nat Ecol Evol. 2025 Sep 1;9(10):1781–90. doi: 10.1038/s41559-025-02829-x (PMC12507691; doi:10.1038/s41559-025-02829-x)
Supplement: Supplementary file 1 — Supplementary text, Tables 1–3 and Figs. 1–7. [file 41559_2025_2829_MOESM1_ESM.pdf]

nature ecology & evolution

Supplementary information

https://doi.org/10.1038/s41559-025-02829-x

Hominin glacial-stage occupation 712,000 to 424,000 years ago at Fordwich Pit, Old Park (Canterbury, UK)

In the format provided by the authors and unedited

Supplementary Information

Supplementary Methods

Excavation

Trench One

Excavations in Trench One proceeded following the methods described in Key et al. (2022). Having been expanded to two by three metres at ground level (from the original two 1x1 meter test trenches in 2020 [Key et al., 2022], with the third metre representing the one-metre gap between these early test trenches), the trench increased to approximately three by three metres at its lowest 2.3 m depth, due to the sloped bank of the quarry's edge (Figure 2). Excavated by hand in 10 cm vertical spits, sediment was found to be consistent with previous descriptions (Key et al., 2022) insofar as we continued to see sand lens and roughly stratified variable gravels consistent with deposition through glacial fluvial activity with changeable energy, including loose fine gravels through to highly compacted large gravels that included occasional nodules >10 cm in size. All sediment was passed through 6mm sieves. All lithic items displaying multiple features indicative of their intentional removal from a core by a hominin were recovered, labelled and bagged. These objects were then 'filtered' by the experienced lithic analysts in the author team (AK, TP, MP, CMR, JC, FS) to produce a final assemblage that removed those more likely to be produced through natural fluvial processes. Depending on the year of excavation the individuals analysing the lithics varied but were never below three.

Trench Two

The excavation of Trench Two began with the intention of exposing sediments identified by Bridgland et al. (1998) and dated by Key et al. (2022), but that were absent from Trench One. This trench is two metres southwest (i.e., two metres along the length of the old quarry face) to the southernmost exposure of gravels and sands cut by Bridgland et al. (1998; Briant et al., 2024 [their 'Section 1', c. 10 metres south of 'Section 2']), and is dug into the highest ridge that presents the remnants of Fordwich Pit (note that this does not necessarily reflect the highest point of the gravels originally) (Key et al., 2022). This pit was originally opened to an extent of 1 x 2 m at its superior level and progressed in 10 cm vertical spits, through topsoil, fine sand, and then a sequence of gravels and sands. All sediment below the topsoil was passed through a 6mm dry sieve. While the fine sand (dated to low MIS 12 by Key et al. [2022]) was sterile of artefacts, a low density of clear artefacts, including extremely fresh pieces, were recovered from the initial gravel layers found immediately below it, at a depth of 1.5-1.6 m below the surface.

Trench Two was then expanded to 4 x 3 m (width by length). Topsoil was removed, with horizontal steps subsequently added as the excavation progressed. From a depth of around 1 m, the trench was narrowed to 2 x 3 m, which was dug for a further 1 m. At this point, the excavation focused on a 1 x 2 m section, which reached a depth of 3.5 m, and a final 1x1 m square that reached a maximum depth of 4.6m below the surface. Artefacts were again found at low density across a horizontal extension at 1.5 - 1.6 m depth, with some artefacts located at 1.8 to 2.0 m, as well as one probable flake at the very bottom of the upper gravels (3.4 - 3.5 m). The entirety of this 2 m Upper Gravel sequence was consistent with the sands, gravelly-sands, sandy-gravels, and gravels cut by Bridgland et al. (1998) and further documented by Key et al. (2022).

At around 3.5 m depth, the Middle Sand Layer (see Bridgland et al., 1998 and Key et al., 2022; Extended Data Figure 10) was clearly identified in section, at a maximum thickness of around 15cm in the North Section, but thinning to the point of non-detection in the South Section. This layer marks the transition from the Upper Gravels to the Lower Gravels, and the shift from MIS12 to MIS16/17 sedimentation. We found one flake artefact in situ in the upper part of the lower gravels, as documented in Figures 2 and 3, at a depth of 3.6-3.7 m. However, additional flakes would be beneficial from this depth to conclude the presence of artefacts with absolute certainty.

Sediment throughout the section ranged from compacted flint clasts 3-7 cm in size (rarely inclusions would reach >10 cm), to pockets of loose fine gravel, fine grain sand lens, and sand layers. These data are all consistent with a glacial river system, sealed at the top by the glacial MIS 12 fine sand. The lithic analysis procedure described for Trench One was repeated for all objects recovered from Trench Two.

Trench Three

Located midway between Trenches One and Two (c. 50 m north of Trench Two), Trench Three measured 3 x 2 m, and proceeded to a depth of 1.8m in the southern part of the trench. On the southern edge, around 130 cm of topsoil was followed by 15-30 cm of fine sand and then gravel, of which we excavated 35 cm. The northern edge of the trench was characterised by 100-110 cm of topsoil followed by around at least 60 cm of gravel. Although no dating was undertaken in this trench, we are confident in interpreting these excavated gravels as the Upper Gravels identified in Trench Two as they are located beneath the only known fine sand on the site and biface thinning flakes are present. Artefacts were located within the first 50 cm of the gravel, but predominantly the first 20 cm. Rolling was limited-moderate on all of the artefacts discovered, indicating relatively minor fluvial transport.

Test Trenches and Exposures

In addition to the three excavated trenches, seven test trenches and four exposures were machine dug to increase understanding of the site, its remaining sediment's stratigraphy, and the distribution of artefacts through these sequences (Supplementary Figures 1 and 2). These trenches and exposures ranged from the north-easterly limit of the remaining intact quarry edge through to its south westerly periphery. Two exposures revealed that within 10 metres of Trench One, in the northwesterly direction, the gravels increase substantially in depth (upwards of 6 metres) (Extended Data Figure 1). This rapid increase in sediment depth is consistent with the gravel depth increasing substantially once the brow of the Lambeth Group sands is reached as you move into the present Stour valley, potentially supporting the interpretation of these gravels as having been redeposited after an earlier deposition. One further exposure c. 5 metres west of Trench One revealed the stratigraphy identified in the excavated trench to continue at a broadly consistent level. Additional test trenches were dug in northern, northeastern, and western locations relative to Trench One (Supplementary Figure 1). Those to the north and northeast identified deep, variable gravels of unknown depth consistent with those excavated at Trench One. Those to the west revealed all gravels to have been removed, with the Lambeth sands reached at a shallow depth, either through the earlier aggregate extraction or later construction work. All test trenches/exposures in the vicinity of Trench One, bar four, revealed flake artefacts at low frequencies, but these were recovered from spoil and cannot be correlated with specific levels. Given the number of exposures and test trenches dug, and the recovery of only flake artefacts, it seems unlikely that handaxes are present at the northwestern edge of the quarry in any great number, and we now (Key et al. 2022) interpret Willock's 1932 (Roe, 1968) statement identifying a higher density of artefacts in the west edge of the pit to refer to the extreme west. Following the creation of these exposures and trenches in the northern portion of the quarry, it has become clear that the

sediments associated with a majority of the artefacts by Smith (1933) accord with the west-to-south-west gravels around Trench Two.

One additional exposure was created three metres to the south of the more southerly Bridgland et al. (1998) exposure. The exposure revealed sediment consistent with the two Bridgland et al. (1998) exposures, and while we recovered a couple of likely flakes from the spoil of the lower MIS 16 gravel, we cannot reliably associate them with a specific depth. Finally, a test trench was created midway between Trench One and Two, 10 m north of Trench Three, as the quarry's edge starts to curve eastwards. Here, the topsoil was substantial in depth (> 1m) and rested directly on fluvial gravels, revealing the fine sand of Trench Two and Three to no longer, or have never, precede(d) along the whole western limit of the quarry. This further supports the location of Trench Two as closer to the discovery site of the majority of artefacts during the 1920s (Smith, 1933; Roe, 1968).

Elevation was recorded from the top of each trench using a Leica Geosystems 1200 Differential GPS system. Results were processed using Leica Infinity software and reported with respects to the Ordnance Survey's OSGM15 geoid model. Note that these new elevation data confirm the two previously noted elevations in Bridgland et al. (1999) to be inaccurate (Extended Data Figure 10).

Museum Store Collections

To help contextualise the newly excavated artefacts, the existing Fordwich Pit material held at the British Museum as part of the Bowes Collection was analysed by JC. In agreement with the authors discussed in the main text, the collection is indeed broadly elongated and often thick, which includes tri- and quadrihedral specimens that are completely atypical for the British MIS 15, MIS 13 and MIS 11 Acheulean record (Figure 4; Extended Data Figure 6). There are some unusual crude bifacial forms from the Lower level of La Noira (MIS16), but true pick-like forms are only known from the European record at La Boella (Spain) in the Early Pleistocene sites (Moncel et al., 2013, 2016, 2020; Mosquera et al., 2016; Ollé et al., 2023). These elongated specimens from Fordwich Pit tend to maintain a narrow width along a majority of their length, especially in the lower half, and removals appear targeted towards the tip.

Equally, there is a clear and sizeable sample of more ovate specimens that are widest around the midpoint of length, and the clear presence of soft hammer flaking, including *tranchet* removals (Figure 4; Extended Data Figure 6). Indeed, the thickness/width (refinement) ratios from the complete assemblage show two distinct peaks (Extended Data Figure 5), supporting two distinct assemblages of handaxes being present at the site. The presence of *tranchet* flaking on multiple more-ovate artefacts is particularly interesting because it is a clear technological marker of biface use in the British MIS 13 record, especially from Boxgrove (Roberts and Parfitt, 1999), and would align with the aforementioned second, later occupation at Old Park. A couple of these more heavily flaked artefacts are also made on banded flint of the same type as some flakes recovered from the top of the Upper Gravels dated to MIS 12, while other ovate bifaces on this banded material are patinated to a colour and pattern that is strikingly similar to these flakes. While all the artefacts in the Fordwich collection tend to be quite patinated (though only minimally-moderately rolled), this banded flint is not present amongst the thicker, elongated aspects of the pre-existing Fordwich Pit handaxes.

2D geometric morphometric analysis of handaxe shape was also employed to compare a subset of the old handaxe collections from Fordwich pit (n = 65) to other relevant European Acheulean assemblages (n =488). The European comparative sample largely derives from the openly-accessible dataset published by Clark et al. (2024), to which we have added the unusually-early picks from Barranc La Boella, as published by Ollé et al. (2023), due to the unusually crude forms included within the Old Park collections. Together,

these comparative assemblages cover the late Early Pleistocene and early Middle Pleistocene from southern, central, and northern Europe (Barranc La Boella, Notarchirico, La Noira, Moulin Quignon, Brandon Fields, and Maidscross Hill), and extend throughout the Middle Pleistocene of Britain and France. The analysis followed the methods described in Clark et al. (2024), in which plan-view photographs of artefacts were converted to silhouettes and 60 equidistant coordinates were generated in tpsDig2.31. These were imported into PAST3, where they were subject to a Generalised Procrustes Analysis, and subsequently an Elliptical Fourier Principal Component Analysis (PCA) on the first 30 Fourier harmonics.

Only Principal Components (PCs) 1-3 account for more than 5% of shape variance. PC1 represents a measure of elongation and tip shape, with greater values associated with longer artefacts relative to width and more pointed tips and lower values associated with shorter objects relative to width and more rounded tips, while PC3 represents the position of maximum width, with higher values associated with this being closer to the base of the artefact and smaller values associated with this being closer to the tip. PC2 is a measure of handaxe symmetry, and thus we follow Shipton et al. (2023) in plotting PC1 against PC3 to better reflect specific changes in overall shape.

Comparisons of handaxe shape between Old Park and other European sites are shown in Supplementary Figure 5. These data show that the Old Park artefacts are overall unusually elongated for European bifaces, and divergent from the earliest definitive European Acheulean assemblages documented further south in the continent (La Noira and Notarchirico). The picks from Barranc La Boella cover almost the entire range of shape variation, making it hard to determine the extent of control over outcome shape in this assemblage. This seems to be in contrast to the much tighter limits of variation at Old Park; although Old Park is entirely within the shape range of Barranc La Boella. The Old Park handaxes could be said to compare more favourably with early British assemblages (Brandon Fields and especially Maidscross Hill), but see lower PC1 variation and are still divergent. The Old Park handaxes are clearly distinct from Warren Hill, regardless of wear at that site (see Moncel et al., 2015), as well as from the British MIS13 sites of Boxgrove and High Lodge. In comparison with MIS11 assemblages, Old Park is again distinct from all except Saint Acheul, but this site includes enormous variability, meaning it subsumes almost all included assemblages, and extends much further into negative PC1 variation.

A Shapiro-Wilk test suggests that PC1 values from Old Park are not normally distributed ($W = 0.927$, $p < 0.001$), something that is only shared with Brandon Fields and Elveden at $\alpha = 0.05$ and $n \geq 15$. As shown in Supplementary Figure 6, this non-normality seems to be related to a large peak of PC1 scores around 0.12, a possible second peak around 0.02 (i.e., a bimodal distribution), and an extremely long tail of negative PC values. This may suggest there is more than one population input to the Old Park assemblage, as also hinted at by the unusual peak in the distribution of refinement values above. Furthermore, refinement is significantly associated with PC1 at Old Park ($r = 0.451$, $p < 0.001$), such that thicker artefacts relative to width are also more elongated and pointed. At $\alpha = 0.05$ and $n \geq 15$, only Maidscross Hill, Broom Pits, and Cuxton share a site-level relationship in the same direction, while the corresponding relationship across the European sample is extremely weak and is only significant because of sample size ($r = 0.166$, $p < 0.001$). This relationship between refinement and this measure of plan-view shape at Old Park does not seem to be the product of a reduction trajectory from more elongated and thicker artefacts to less elongated and thinner ones, given there is no relationship between PC1 and either the absolute number of flake scars ($r = 0.005$, $p = 0.971$), or the density of flake scars relative to artefact weight$^{1/3}$ ($r = -0.155$, $p = 0.222$). This latter relationship is absent in spite of a significant association between scar density and refinement ($r = -0.348$, $p = 0.005$).

To investigate this relationship further, the Old Park sample was successively split by the Median, Lower Quartile, and Upper Quartile of refinement, with PC1 compared between these subsets (Supplementary

Figure 7). As not all groups were normally distributed, Mann-Whitney U-Tests were used to evaluate these possible shape differences. Each comparison returned a significant difference between the subsets at $\alpha = 0.05$, with the groups of lower refinement values (meaning thinner artefacts relative to width) having lower PC1 scores (meaning shorter artefacts relative to width and more rounded tips). The PC1 values of these lower refinement value groups are intermediate between those of the higher refinement value groups (meaning thicker artefacts relative to width) from Old Park and the entire distribution of PC1 scores at High Lodge, but are largely distinct from both.

It is noteworthy that to date we have not identified any unmodified banded flint nodules within the fluvial gravels at the site, but it is known to be eroding from the Kent Downs (chalkland) located a few miles from the site, hinting at the anthropogenic transportation of raw materials. Indeed, our overall impression of the unmodified nodules of flint within the ancient Stour river system is one of irregularly formed blocks with multiple inclusions and fractures that tend to be smaller and less homogenous than those recoverable a short distance away on the Kent Downs. We fully expect there to be *some* banded flint within the ancient river bed, but at present it appears to be at low frequencies.

Infrared-Radiofluorescence (IR-RF) Dating

Infrared-radiofluorescence (IR-RF) is, similar to luminescence dating, a method to determine the last sunlight exposure of K-feldspar grains (e.g. Krbetschek et al. 2000; Erfurt et al. 2000; 2003; Erfurt and Krbetschek 2003a). A summary of the research history and currently made methodological progress was recently published in Murari et al. (2021). The method works fully independently from electron-recombination centres and is based on an emission at around 870 nm which relates to the process of electron trapping. Hence, the IR-RF emission is measured while the sample is exposed to a radioactive source. The probability of electron trapping per time decreases with irradiation time. Hence, the most intensive natural RF signal is expected for young samples with a high number of available traps.

The IR-RF signal is relatively hard to bleach and requires several hours of light exposure and saturates at about 1200-1500 Gy (Erfurt and Krbetschek, 2003b). Therefore, the method is recommended for samples in the higher dose range (not Holocene or late Weichselian) and several studies have already demonstrated the applicability of IR-RF for samples beyond the last glacial cycle (e.g. Wagner et al. 2010, Lauer et al. 2011).

The sample preparation and IR-RF equivalent dose (De) measurements conducted were identical to the approach recently published by Key et al. (2022). This is mandatory for a robust comparison of the IR-RF ages. Hence, the K-feldspar preparation, included the common steps of sieving and destruction of carbonates and organic matter using 10 % HCL and 15 % H_2O_2. To extract the K-feldspar from other minerals, the flotation technique (Mialler et al. 1983), followed by a separation with sodiumpolytungstate at a density of 2.58 g/cm3 was used.

All equivalent dose measurements were conducted on a Lexsyg research system at the MPI EVA in Leipzig, Germany, equipped with a calibrated beta ring-source delivering a dose rate of approximately 0.05 Gy/s. The ring source was calibrated with Risoe calibration quartz dosed with 4.81 Gy. The IR-RF signal was measured by a photomultiplier-tube (Hamamatsu H7421-50) which is positioned directly on top of the aliquot. The IR-RF emission was filtered through a Chroma D850/40 interference filter and a plan-convex lens.

Extended Data Figure 7 shows a representative decay curve of the natural and regenerated IR-RF signals which were obtained following the improved IRSAR protocol presented by Frouin (2017). Due to the limited amount of coarse grain K-feldspar available, only 3 aliquots per sample could be measured. We used large (5 mm) aliquots (90-250 µm) for all measurements, hence assuming a strong averaging effect. Consequently, incomplete bleaching of the IR-RF signal could not be detected. As the IR-RF signal is characterised by a hard to bleach component, potential age overestimation has to be taken into account, theoretically even for the youngest ages returned. As the samples were taken in close proximity to each other, a similarity in age is assumed for the samples investigated.

The dose rate (concentration of K, Th, U) was determined using high resolution gamma spectrometry at the VKTA Dresden. The specific activity of 238U was calculated based on the specific activity of 234Th. 214Pb and 214Bi were used for 226Rn, 228Ac for 228Ra and 212Pb and 208TI to get the activity of 228Th. For IR-RF age calculation an a-value of 0.067 ± 0.012 was used (Kreutzer et al. 2018). For internal dose rate, a K-content of 12.5 ± 0.5 % was used (Huntley and Baril 1997). Dose rate conversion factors were taken from Guérin et al. (2011) and the cosmic dose rate was calculated based on Prescott and Hutton (1994). A water content of 20 ± 10 % was used. The high error was chosen with respect to the uncertainties of the water content over the relevant geological time scale. The cosmic dose contribution was calculated following Prescott and Hutton (1994) by considering the elevation above sea level as well as the geographical position (longitude and latitude) and the burial/ sampling depth. As the samples were taken in very close proximity to each other, all cosmic dose contributions are at approximately 0.17 ±0.02 Gy/ka.

Palaeomagnetic Analyses

For palaeomagnetic analyses an orientated hand sample (12x8x5 cm) was sampled for Alternating Field (AF) and Thermal (TH) demagnetisation. At the Fort Hoofddijk Paleomagnetic Laboratory (Utrecht University, the Netherlands) five orientated specimens were taken from the hand sample for analysis. Specimens for AF and TH demagnetisation were sampled by gently pushing custom-made plexiglass/ quartz containers, with standard paleomagnetic sampling dimensions (25 mm diameter, 22 mm length), into the sediment. Both AF and TH measurements were made using direct current superconducting quantum interference device (DC-SQUID) magnetometers manufactured by the ‘2G’ company (Mountain View California, USA). The instrument sensitivity is 3 ×10^(-12) Am2 and NRM intensities were typically several orders of magnitude higher than the instrument sensitivity. Both magnetometers used for this study are housed in a magnetically shielded room (residual field < 200 nT).

AF demagnetization in 12 steps (0, 5, 10, 15, 20, 25, 30, 40, 50, 60, 70, 80, 100 mT) to up 100 mT was carried out with a robotized system. The robotized interface for sample manipulation was built in-house and up to 96 samples contained in dedicated cubic holders (edge 30 mm) are loaded onto a sample plateau and the robot loads them in batches of eight onto a tray that can be slid through the magnetometer and demagnetization coils (Mullender et al., 2016). After each step the remaining NRM was measured using a different 2G DC-SQUID cryogenic magnetometer. Static three-axial AF demagnetization was done with a so-called in-line AF demagnetization coil set directly attached to the magnetometer. Samples are processed fully automatically with the so-called ‘three position protocol’ that compensates for the magnetic moment of the transport tray. This ensures optimal processing of weakly magnetic samples. Stepwise progressive TH demagnetization of the NRM was performed on one specimen (specimen k1.5) up to a maximum of 260 °Celsius, in nine temperature steps using an ASC thermal demagnetizer (residual field <20 nT).

The results of the demagnetisation were interpreted using principal component analysis (Kirschvink, 1980) to identify the Characteristic Remanent Magnetization (ChRM) directions with a minimum of four consecutive steps (n = 4), after visual inspection of Zijderveld diagrams (Extended Data Figures 8 and 9)

(e.g., Zijderveld, 1967). The directions were calculated online using Paleomagnetism.org, an online open-source multi-platform for paleomagnetic data analyses and data storage (Koymans et al., 2016, 2020). Within the platform a suite of techniques is used to statistically interpret the results (Fisher, 1953; Kirschvink, 1980; Tauxe et al., 2010; Deenen et al., 2011).

Palaeoenvironmental (Phytolith) Data

Samples were processed at Seoul National University's Archaeological Science Lab and analysed in their Archaeobotanical Lab. The extraction protocol follows a modified version of Madella et al. (1998).

Approximately 5g of sediment were weighed out using a 0.000 g accurate scale and placed in 50 ml test tubes before their weights were recorded. 25 ml of 5% concentration hydrochloric acid was added to the samples and they were placed in a hot block at 40°C for one hour. Caps were placed on the tubes but not screwed down to prevent contamination but to allow for any gaseous by products to escape. This stage removes the carbonates. Samples were allowed to cool to room temperature and distilled water was added to bring the liquid contents of the tube to 50 ml. The samples were placed in a centrifuge and run at 1500rpm for 3 minutes. The supernatant liquid was then carefully poured off, leaving the pellet at the base of the tube. The sample was then topped to 50 ml again with distilled water, and the centrifuge and pouring stage repeated until a further 3 rinses were complete. On the final rinse the samples were topped to 50 ml with a 5% solution of sodium hexametaphosphate, capped and shaken vigorously. They were then left to settle overnight. This stage is to deflocculate the clays. The samples were then carefully placed in the centrifuge and spun at 1500 rpm for 3 minutes. The supernatant was poured off, leaving the pellet, and distilled water to the 50 ml level added. Centrifugation and rinsing was repeated a further 3 times. After the final rinse 15ml of 30%w/v hydrogen peroxide was added to the samples and they were placed in the hot block at 30°C for 4 hours. This stage was to remove organics and the caps were placed on the tubes but not screwed down as in the hydrochloric stage. Samples were allowed to cool to room temperature and then topped with distilled water to 50 ml. The samples were then carefully placed in the centrifuge and spun at 1500 rpm for 3 minutes. The supernatant was poured off, leaving the pellet, and distilled water to the 50 ml level added. Centrifugation and rinsing was repeated a further 3 times.

On completion of this stage the AIF (acid insoluble fraction) samples were placed in the oven at 60°C to dry completely. Once dry they were weighed and the sample weight (minus tube and cap starting weight) recorded. 20 ml of the heavy liquid sodium polytungstate with a specific gravity of 2.35 g/cm3 was added to the AIF samples and they were agitated gently to ensure the liquid permeated the entire sample. The sample was placed in the centrifuge at 1500 rpm for 3 minutes then carefully removed. A floating ring of phytoliths were then present, and these were collected and transferred to fresh labelled 50 ml tubes. The AIF samples were centrifuged against 1500 rpm for 3 minutes and the collection repeated. Into the new tubes with the phytoliths distilled water was added to the level of 50 ml and the sample vigorously shaken. The tubes containing the remaining AIF were then sent for sodium polytungstate recycling. The phytolith samples were then centrifuged at 2000 rpm for 5 minutes, and the supernatant water poured carefully off. This was repeated a further 4 times to ensure the samples were clean and free of the heavy liquid. After the final rinse the phytoliths were pipetted into 10 ml weighed vials and put in the oven at 60°C to dry. Once dry the weight of the phytoliths (minus the vial and cap) was recorded.

Due to the low quantities of phytoliths recovered it was determined that temporary slides were more appropriate than permanent slides. This would allow for objects to be moved, turned, and increase the chance of identification, something not possible in permanent mounting solution. Slides were made using a small (unmeasured due to testing for phytolith density in the first instance) quantity of phytolith powder

placed on a cleaned slide with a few drops of microscopy immersion oil added. The material and oil were mixed using a cocktail stick and a cover slip dropped on top. This was secured with clear nail polish. The decision to not make permanent slides was determined to have been important as it allowed better options to see the morphotypes, and the lack of weighing the material added did not affect the analysis as there were so few phytoliths present quantification would not have been possible even if multiple slides would have been made (estimate average is 10-20 phytoliths per slide, and 250-350 needed for a quantifiable analysis).

Identification was made using a Leica DM750 microscope, and morphotypes name using the ICPN 2.0 (International Committee for Phytolith Taxonomy (ICPT) et al., 2019).

Environmental Suitability for Acheulean Populations

The estimates of environmental suitability for hominin populations at Old Park (Figure 1) have been extracted from the palaeoecological models presented in Leonardi et al. (2024a). The relevant palaeoclimatic variables for the coordinates of the site were extracted with the R package *pastclim* (Leonardi et al. 2023) using the Krapp et al. (2021) reconstructions. This resulted in a time series of each climatic variable estimated every 1,000 years for the period between 712 – 424 thousand years BP (i.e. from the start of MIS 17 to end of MIS 12). The R package *tidysdm* (Leonardi et al 2024b) was then used to project them over the results of the palaeoecological models performed in Leonardi et al (2024a), to get precise estimates of the suitability for Old Park in the period of interest.

Summary: Bringing Data Together

Here we report on the Lower Palaeolithic occurrence of Old Park for the first time. Artefacts have previously been described from Fordwich Pit, which is located on the eastern edge of the Old Park and Chequers Wood Site of Special Scientific Interest, but Lower Palaeolithic artefacts have now been discovered eroding from gravel terraces more widely across the site. These terraces are at a similar ordnance datum to those observed in Fordwich Pit, and are therefore likely to be of a similar age. Moving forward, Fordwich Pit should be viewed as part of the wider Old Park Palaeo-fluvial landscape. This does not detract from the importance of Fordwich Pit as a named site, nor its role in the epistemology of British Palaeolithic archaeology, but it does recognise the wider landscape's archaeological and geological importance, and the widespread evidence of Middle Pleistocene hominin presence in the area. A fact all the more important given the scarcity of similarly aged artefact-bearing sediment in Northern Europe.

Since 2020, three large excavated trenches, seven test trenches and four exposures have been created. Nonetheless, we have covered only a fraction of the total area of the original quarry, and it is not surprising that handaxes have not yet been recovered (Supplementary Figure 1). If only 330 handaxes were historically recovered from a quarried area roughly 300 × 150 m in size, the density of artefacts will be low (although it is likely many more handaxes were not recovered). For every handaxe produced by Pleistocene hominins, a large number of flakes would, however, have been created, in addition to those created via flake and core knapping processes, explaining the flake artefacts found to date. Importantly, flakes from the superior portion of the MIS 12 gravel in Trenches 2 and 3 were likely removed during the production of handaxes. An inference proposed by us based on their form and technical attributes, but also supported more widely by colleagues specialising in Northern European Lower Palaeolithic archaeology (e.g., Ashton,

N. pers. comm). Although it is important to note that the number of handaxe thinning flakes varies between analysts, which is to be expected given their subjective designation.

It is, however, the stratigraphic location of these and other artefacts, including those collected historically, that form the focus of the present article. Four clear sedimentary units are present at Fordwich Pit. The youngest and most superior is a ~120 cm deep section of largely homogenous fine sand present in Trench Two, which reduces to ~ 20 cm by the southern edge of Trench Three, and is dated via two IR-RF samples to 379 ±21 ka (MIS 11) and 455 ±24 ka (MIS 12). Although these two ages vary, these sands are best interpreted as being from a low energy fluvial environment (although we can't entirely rule out aeolian processes) linked to the MIS 12 glacial period. Two samples of these sands were investigated by DB, with both found to be fine-medium, subangular / subrounded, and typically ~0.2mm in size. The upper sample had traces of a brown, probably ferruginous matrix, while the lower had traces of a non-calcareous (tested via HCl reaction) pale matrix. Overall, these fine sands are interpreted to be reworked Palaeogene sand, likely from the local Thanet or Lambeth Group sands.

Immediately beneath the fine sand, deep fluvial gravels (160 cm) also dated to MIS 12 via three highly consistent IR-RF samples (423 ±29, 433 ±23, and 437 ±29 ka) have been demonstrated to contain flake artefacts, some of which are very fresh and display little to no evidence of being moved within a fluvial river system. Moreover, almost all flakes, including the handaxe thinning flakes, were recovered from the first 20 cm of these gravels. Potentially, the (now) small tributary of the river Stour that runs parallel to the site and c. 30 m from Trench One, which has incised a small valley immediately next to Fordwich Pit, may have moved these gravels and the fine sand sediments from elsewhere during MIS 12, potentially when it had more energy, explaining how they overlay earlier MIS 16 deposits (see below). Together, this evidence could be interpreted as hominins knapping on an exposed, previously fluvially-deposited gravel bank during the MIS 12 glacial period, prior to the superior fine sands being deposited during the same cold stage. In effect, this demonstrates the presence of Middle Pleistocene Acheulean hominins in southern Britain during the Anglian (MIS 12) glacial period.

Beneath the MIS 12 gravels in Trench Two, and potentially in Trench Three (excavations did not extend to this depth in Trench Three), are 220 cm of fluvial gravels and sands dated via IR-RF to 607 – 826 ka (607 ±45, 643 ±78, 780 ±39, 795 ±43, and 826 ±40 ka). While the dates are variable, it is important to note that none display range overlap with the more superior sediments or those identified elsewhere (Figure 2). The two younger dates from the lower gravels are associated with early MIS 15 and mid-MIS 16 and link the deposition of the gravels to MIS 16 fluvial activity, which is supported by the formation of substantial fluvial gravel depositions strongly associated with glacial stages (Van Huissteden et al., 2013; Bridgland, 2021). The three earliest dates may reflect the use of the method at the upper end of its functional (temporal) range or an overestimation due to incompletely bleached IR-RF signals caused by rapid transportation and burial of sediment. An inference supported by the palaeomagnetic analyses, which identified the Brunhes normal polarity, confirming their deposition less than c. 773 + 2 ka. Thus, we interpret these lower gravels to be derived from MIS 16 fluvial activity, which is consistent with earlier fluvial incision and uplift estimates by Bridgland et al. (1998) and the sedimentary aggradation interpretation presented in Supplementary Figure 2. As the IR-RF signal is characterised by a hard to bleach component, overestimation for the 607 and 643 ka dated samples also needs to be taken into account. We do, however, consider these MIS 16 dates as representative of the sediment's deposition given: the consistency of results and lack of range overlap returned for other layers, the aforementioned incision and uplift estimates, the next youngest period of potential fluvial aggradation (i.e., MIS cold stage) being represented elsewhere on the site by a separate cluster of dates, and the presence of handaxe forms atypical for MIS 15-11 (see below). Previous flake discoveries by Mark White and colleagues, which appear to be from these gravels (Bridgland et al., 1998;

Briant et al., 2024), along with our own discovery of two seemingly knapped flakes from this depth, could then be interpreted as evidence of hominin presence during either MIS 16 and/or MIS 17.

The presence of both MIS 12 and MIS 17/16 artefact-bearing sediments on the western edge of Fordwich Pit near the brow of the hill, where the majority of the historically recovered handaxes were recovered (Smith, 1933; Wymer, 1977; Roe, 1968), presents the possibility that these bifaces were collected from both stratigraphic units, and in turn, discarded by two temporally distinct hominin populations. This inference is supported by the technologically and morphologically variable assemblage of handaxes known from the site. Indeed, there are highly elongated forms that are poorly worked with few flake removals and quite unlike northern European handaxe assemblages associated with MIS 15 to 11. This includes the presence of pick-like forms only known from Early Pleistocene European sites (Mosquera et al., 2016; Ollé et al., 2023), and crude forms more typical of early Middle Pleistocene Acheulean assemblages in Northern Europe (Moncel et al., 2013, 2016, 2020, 2022; Antoine et al., 2018). Equally, there are some heavily flaked and highly worked handaxes, some of which display *tranchet* flake removals, that are consistent with the thinning flakes recovered from the MIS 12 sediment and other British MIS 13/12 biface assemblages. Notably, some of these more heavily shaped historically collected handaxes were created using the distinctive banded flint also seen in some of the newly excavated MIS 12 flakes. Indeed, the attribution of these later handaxe forms to the MIS 12 sediment appears secure. The best-fit scenario for the explanation of the technological and morphological diversity in the handaxe assemblage is that they derive from two quite distinct populations of hominins, which we can now infer were separated in time by several hundred thousand years. The MIS 16/17 presence of handaxes at Old Park would make these the earliest known handaxes in northern Europe, while the MIS 12 handaxes demonstrate the repeated occupation of Old Park by Acheulean hominins.

Phytoliths were recovered from four sediment samples collected in the MIS 12 layers of Trench Two, with three located in the upper gravels and the fourth located in the lower limit of the fine sand. Notably, two of these samples came from immediately above, or from within, the upper 20 cm of the upper gravel which contained the majority of MIS 12 artefacts. From all samples, the identifiable phytoliths were grass or dicot morphotypes, with little evidence of other vegetation. The emergent ecological picture is consistent with an open, northern European grassland habitat during a glacial period. While caution is, of course, required when interpreting these environmental data – particularly given the poor preservation of the phytoliths - if reworked phytoliths from more superior sediments had migrated to these lower sampled levels, as could be argued, we may also have expected to not only identity tree and shrub morphotypes from later (e.g., MIS 11 or 9) periods, but also observed variation in phytolith preservation. Neither of which occurred. Moreover, the grass short cells that were predominantly observed are noted to be relatively stable in shape over prolonged periods, and are unlikely to be selectively sorted by water movement within the sediment (see online methods). Thus, present palaeoenvironmental data is tentative, but supports the presence of hominins in a predominantly grassland environment during a period of MIS 12, be it all year-around or only during the summer. Indeed, the palaeoecological models created here indicate that during the MIS 16, 14 and 12 glacial stages, Old Park would likely have displayed an environment suitable for hominin habitation, albeit with periods that were only occupiable during the summer or would have been classed as on the periphery of a more suitable ‘core’ habitable area (Figure 1).

Trench One is located at the northern edge of the quarry and has returned an assemblage of principally flake artefacts first reported by Key et al. (2022). In Supplementary Figure 3 additional artefacts from the 2021-2023 excavations are reported, and there is no doubt in the hominin presence evidenced in these sediments, even if past taphonomic (fluvial) processes make some artefacts hard to distinguish or have created flint flakes with features suggestive of an anthropomorphic origin (Supplementary Figure 4). Previously, these Trench One sediments were dated via IR-RF to MIS 10/11 (347 ±22, 372 ±22, 375 ±22,

385 ±21, 383 ±21 ka) and MIS 14 (570 ±36 and 513 ±30 ka), with the younger more superior dates suggested to result from substrate gullying and solifluction, and the MIS 14 dates to reflect the sediments original deposition (Key et al., 2022). This is still one interpretation, with the quarry possibly retaining gravels deposited in MIS 16, 14 and 12. Given the location of Trench One at the northern edge of the quarry at the edge of the valley, along with the near-identical vertical alignment of these gravels and the lower gravels in Trench Two (Supplementary Figure 2), it is however also possible that the MIS 14 dates also result from gullying and solifluction actions after the gravels were first deposited in MIS 16. Indeed, given that the Trench One sediments directly overlay the Lambeth Group sands found beneath the MIS 16 gravels in Trench Two, the latter may represent a more parsimonious explanation, with these MIS 16 gravels in turn being found across the whole site (Supplementary Figure 2). In turn, the flake and other artefacts in Trench One could also be derived from MIS 16/17.

Taken together, it is likely that the banks of the ancient Stour river were repeatedly occupied by hominin populations during the mid-Pleistocene. Old Park likely preserves rare evidence of hominin presence in northern Europe from MIS 17–16 (712 - 621 ka), during MIS 13-12 – potentially on two occasions, but at least once – and possibly also during MIS 15–14 (563 - 533 ka) depending on one's aggregation interpretation of Trench One. The MIS 12 sediment is important for its evidence of high-latitude Anglian-stage occupation by mid-Pleistocene Acheulean hominins. Phytolith data suggests a grassland environment with mixed grasses and flowering plants. The MIS 17–16 dated gravels are significant for their association with the substantial, but technologically and morphologically varied, handaxe assemblage recovered in the 1920s. The rougher, more irregularly flaked and elongated handaxes in this assemblage potentially represent the earliest known handaxes from northern Europe, while the more heavily flaked forms may reveal a reoccupation by Acheulean populations ca. 200,000 years later.

Supplementary Information References

As, J.A., Zijderveld, J.D.A., 1958. Magnetic cleaning of rocks in palaeomagnetic research, Geophys. J. R. Astron. Soc. 1: 308–319

Briant, R.M., Whiteman, C.A., Haggart, B.A., Bridgland, D.R., Egberts, E., Grant, M.J., Knowles, P.G., Schreve, D.C., Toms, P.S., Wenban-Smith, F.F., White, M.J. 2024. Quaternary rivers, tufas and mires of Southern England: description of Geological Conservation Review sites. *Proceedings of the Geologist's Association.*

Bridgland DR, Keen DH, Schreve DC, White MJ. 1998 Summary: dating and correlation of the Stour sequence. In The Quaternary of Kent and Sussex (eds Murton JB, Whiteman CA, Bates MR, Bridgland DR, Long AJ, Roberts MB, Waller MP). London, UK: Quaternary Research Association

Bridgland D.R. 2021. The role of geomorphology in the Quaternary. In: *The history of the study of landforms or the development of geomorphology. Volume 5: geomorphology in the second half of the twentieth century* (eds Burt TP, Goudie AS, Viles HA). London, UK: Geological Society.

Cabanes, D., Weiner, S., Shahack-Gross, R., 2011. Stability of phytoliths in the archaeological record: a dissolution study of modern and fossil phytoliths. J. Archaeol. Sci. 38, 2480–2490.

Deenen, M.H.L., Langereis, C.G., van Hinsbergen, D.J.J., and Biggin, A.J., 2011. Geomagnetic secular variation and the statistics of palaeomagnetic directions. Geophys. J. Int. 186: 509–520.

Erfurt G, Krbetschek MR, Trautmann T, W. Stolz W, 2000. Radioluminescence (RL) behaviour of Al2O3:C-potential for dosimetric applications. *Radiation Measurements* 32(5-6): 735-739

Erfurt G and Krbetschek MR, 2003a. IRSAR - A single-aliquot regenerative- dose dating pro-tocol applied to the infrared radiofluorescence (IR-RF) of coarse-grain K feldspar. *Ancient TL* 21: 21-28.

Erfurt G and Krbetschek MR, 2003b. Studies on the physics of the infrared radioluminescence of potassic feldspar and on the methodology of its application to sediment dating. *Radiation Measurements* 37(4-5): 505-510

Erfurt G, Krbetschek MR, Bortolot VJ and Preusser F, 2003. A fully automated multi-spectral radioluminescence reading system for geochronometry and dosimetry. *Nuclear Instruments and Methods in Physics Research Section B* 207(4): 487-499

Fisher, R., 1953. Dispersion on a sphere. Proc. Roy. Soc. Lond. A 217: 295–305

Frouin M, Huot S, Kreutzer S, Lahaye C, Lamothe M, Phillippe A, Mercier N. 2017 An improved radiofluorescence single-aliquot regenerative dose protocol from K-feldspars. Quat. Geochronol. 38, 13–24.

Guérin, G., Mercier, N., Adamiec, G., 2011. Dose-rate conversion factors: update. Anc. TL 29, 5–8.

Huntley, DJ, and Baril, MR. 1997. The K content of the K-feldspars being measured in optical dating or thermoluminescence dating. Ancient TL, 15.

Prescott, J.R., and Hutton, J.T., 1994. Cosmic ray contributions to dose rates for luminescence and ESR dating: large depths and long-term time variations. Radiat. Meas. 23, 497–500.

International Committee for Phytolith Taxonomy (ICPT), Neumann, K., Strömberg, C.A.E., Ball, T., Albert, R.M., Vrydaghs, L., Cummings, L.S., 2019. International Code for Phytolith Nomenclature (ICPN) 2.0. Ann. Bot. mcz064. https://doi.org/10.1093/aob/mcz064

Key, A., Lauer, T., Skinner, M.M., Pope, M., Bridgland, D.R., Nobel, L. and Proffitt, T. 2022. On the earliest Acheulean in Britain: first dates and in-situ artefacts from the MIS 15 site of Fordwich (Kent, UK). Royal Society Open Science 9: 211904

Kirschvink, J., 1980. The least-square line and plane and the analysis of paleomagnmetic data. Geophys. J. Int. 62: 699–718.

Koymans, M.R., Langereis, C.G., Pastor-Galan, ´ D., and van Hinsbergen, D.J.J., 2016. Paleomagnetism.org: An online multi-platform open-source environment for paleomagnetic data analysis. Comput. Geosci. 93: 127–137.

Koymans, M.R., Hinsbergen, D.J.J., Pastor-Galan, ´ D., Vaes, B., and Langereis, C.G., 2020. Towards FAIR Paleomagnetic Data Management Through Paleomagnetism.org 2.0. Geochem. Geophy. Geosy. 21: e2019GC008838.

Krapp, M., Beyer, R.M., Edmundson, S.L., Valdes, P.J. and Manica, A. 2021. A statistics-based reconstruction of high-resolution global terrestrial climate for the last 800,000 years. Scientific Data 8: 228

Krbetschek MR, Trautmann T, Dietrich A and Stolz W, 2000. Radioluminescence dating of sediments: Methodological aspects. *Radiation Measurements* 32(5-6): 493-498, DOI 10.1016/S1350-4487(00)00122-0.

Kreutzer, S., Schmidt, C., Fuchs, M. C., Dietze, M., Fischer, M., & Fuchs, M. (2012). Introducing an R package for luminescence dating analysis. Ancient TL, 30(1), 1-8.

Kreutzer S, Martin L, Dubernet S, Mercier N. 2018 The IR-RF alpha-efficiency of K-feldspar. Radiat. Meas. 120, 148–156. (doi:10.1016/j. radmeas.2018.04.019)

Lauer T, Krbetschek M, Frechen M, Tsukamoto S, Hoselmann C, Weidenfeller M. 2011 Infrared radiofluorescence (IR-RF) dating of middle pleistocene fluvial archives of the Heidelberg Basin (Southwest Germany). Geochronometria 38, 23–33. (doi:10.2478/s13386-011-0006-9)

Leonardi, M., Hallett, E.Y., Beyer, R., Krapp, M. and Manica, A. 2023. *Pastclim* 1.2: an R package to easily access and use paleoclimatic reconstructions. *Ecography* 3: e06481

Leonardi, M., Lycett, S.J., Manica, A. and Key. A. 2024a. The Acheulean niche: climate and ecology predict handaxe production in Europe. doi: 10.1101/2024.07.19.604259

Leonardi, M., Colucci, M., Pozzi, A.V., Scerri, E.M.L., and Manica, A. 2024b. tidysdm: leveraging the flexibility of tidymodels for species distribution modelling in R. Methods in Ecology and Evolution 15 (10): 1789-1795

Madella, M., Lancelotti, C., 2012. Taphonomy and phytoliths: A user manual. Quat. Int. 275, 76–83. https://doi.org/10.1016/j.quaint.2011.09.008

Madella, M., Powers-Jones, A.H., Jones, M.K., 1998. A simple method of extraction of opal phytoliths from sediments using a non-toxic heavy liquid. J. Archaeol. Sci. 25, 801–803.

Miallier D, Sanzelle S and Fain J, 1983. The use of flotation technique to separate quartz from feldspar. *Ancient TL* 1: 5-6.

Mullender, T. A. T. *et al.* 2016. Automated paleomagnetic and rock magnetic data acquisition with an in-line horizontal '2G' system. *Geochemistry Geophys. Geosystems* **17**, 3546–3559

Murari MK et al. 2021 Infrared radiofluorescence (IR-RF) dating: a review. Quat. Geochronol. 64, 101155.

Prescott, J. R., & Hutton, J. T. (1994). Cosmic ray contributions to dose rates for luminescence and ESR dating: Large depths and long-term time variations. *Radiation Measurements*. https://doi.org/10.1016/1350-4487(94)90086-8

Roe, D.A. 1968. British Lower and Middle Palaeolithic handaxe groups. Proceedings of the Prehistoric Society, 34: 1-82

Smith, R.A. 1933. Implements from high-level gravels near Canterbury. Proceedings of the Prehistoric Society of East Anglia, 7: 165-170

Tauxe, L., Butler, R.F., Van der Voo, R., Banerjee, S.K., 2010. Essentials of Paleomagnetism. University of California Press, California.

Van Huissteden K., Vandenberghe J., Gibbard P.L., Lewin J. 2013. Periglacial fluvial sediments and forms. In: *The encyclopedia of Quaternary science 3* (ed. Elias SA), pp. 490-499. Amsterdam, The Netherlands: Elsevier.

Wagner, G.A., Krbetschek, M., Degering, D., Bahain, J.J., Ahao, Q., Falgueres, C., Voinchet, P., Dolo, J.M., Garcia, T. and Rightmire, G.P. 2010. Radiometric dating of the type-site for Homo heidelbergensis at Mauer, Germany. Proceedings of the National Academy of Sciences 107 (46): 19726-19739

Wagner, G.A., Maul, L.C., Loscher, M. and Schreiber, H.D. 2011. Mauer—the type site of Homo heidelbergensis: Palaeoenvironment and age. *Quaternary Science Reviews* 30 (11-12): 1464-1473

Wymer, J. 1954. Saturday August 21st Fordwich. In: Mepham, L. 2008. The J J Wymer Archive. York: Archaeology Data Service. https://doi.org/10.5284/1000062

Wymer, J. 1977. Fordwich Sat-Sun 30th-31st July 1977. In: Mepham, L. 2008. The J J Wymer Archive. York: Archaeology Data Service. https://doi.org/10.5284/1000062

Zijderveld, J. D. A. 1967. Ac Demagnetization of Rocks: Analysis of Results. In Methods in palaeomagnetism 77 (4): 254-286

Supplementary Information Tables

Supplementary Table 1: Quantities of material recovered for the phytolith analysis.

Sampling Location	Sediment (g)	AIF (g)	Phytoliths (g)
Trench One (542 ka level)	4.9976	4.5774	0.8057
Trench One (542 ka level)	5.0217	4.5987	0.7924
Trench One (542 ka level)	5.0600	4.6190	0.7881
Fine Sand Lowest 10 cm, Trench Two	4.9052	4.1130	0.7896
Fine Sand Lowest 10 cm, Trench Two	5.0589	4.2596	0.8955
Fine Sand Lowest 10 cm, Trench Two	5.0476	4.1252	0.8040
Highest 10cm in Upper Gravel, Trench Two	4.3215	3.8789	0.7956
Highest 10cm in Upper Gravel, Trench Two	4.3975	3.8293	0.8232
Highest 10cm in Upper Gravel, Trench Two	4.3971	3.9035	0.7988
Lowest 10cm in Upper Gravel, Trench Two	4.8882	4.3036	0.7957
Lowest 10cm in Upper Gravel, Trench Two	5.1846	4.4868	0.8229
Lowest 10cm in Upper Gravel, Trench Two	5.0608	4.3785	0.8095
Sand Layer/Lens between Upper and Lower Gravel, Trench Two	4.8264	4.3300	0.7878
Sand Layer/Lens between Upper and Lower Gravel, Trench Two	4.9296	4.3997	0.8099
Sand Layer/Lens between Upper and Lower Gravel, Trench Two	6.2765	5.5850	0.7868

Supplementary Table 2: Plant morphotypes identified in each sample detailed in Supplementary Table 2. ‘P’ demonstrates the presence of the named phytoliths in the relevant sample.

Sampling Location	Grass								Dicot	Indet.
	Generic		Pooideae				Panicoideae	Chloridoideae		
	bulliform flabellate[1]	elongate entire[2]	long-based trapezoid	round-based trapezoid	bilobate-based trapezoid	rondel	bilobate	saddle	spheroid psilate	blocky
Trench One (542 ka level)	p					p		p	p	p
Trench One (542 ka level)	p					p				p
Trench One (542 ka level)				p						p
Fine Sand Lowest 10 cm, Trench Two			p				p	p	p	
Fine Sand Lowest 10 cm, Trench Two			p					p	p	
Fine Sand Lowest 10 cm, Trench Two			p	p	p			p	p	p
Highest 10cm in Upper Gravel, Trench Two									p	
Highest 10cm in Upper Gravel, Trench Two	p								p	p
Highest 10cm in Upper Gravel, Trench Two	p								p	p
Lowest 10cm in Upper Gravel, Trench Two									p	
Lowest 10cm in Upper Gravel, Trench Two									p	p
Lowest 10cm in Upper Gravel, Trench Two	p		p						p	p
Sand Layer/Lens between Upper and Lower Gravel, Trench Two		p								p
Sand Layer/Lens between Upper and Lower Gravel, Trench Two		p								p
Sand Layer/Lens between Upper and Lower Gravel, Trench Two						p				p

[1]could also be Cyperaceae but no other Cyperaceae types were seen. [2] several possible sources, but commonly grass stem.

Supplementary Table 3: Attributes and measurements from artefacts recovered from the Upper Gravel in Trenches Two and Three - see figure for artefact IDs. Dorsal cortex is graded 1 (no cortex), 2 (<50% cortex), 3 (>50% cortex), and 4 (full cortex). Length, width and thickness are taken as 'box measurements' (mm) and weight is recorded in grams.

ID	Type	Breakage	Termination	Scar direction	Dorsal scar count	Platform scar count	Cortex (1-4)	Platform thickness	Max scar length	Length	Width	Thickness	Weight
1	flake	-	feather	proximal	3	2	2	12.6	39.4	58.7	51.7	23.7	50.7
2	flake	-	hinge	proximal	3	1	2	21.7	56.9	65.6	47.1	22.4	21.7
3	flake	crushed platform	feather	-	0	-	3	-	-	58.9	37.3	11.5	20.8
4	core	-	-	proximal	3	2	3	-	43.9	50.1	80.1	55	43.9
5	flake	-	feather	-	0	1	3	10.4	-	68.9	48	32.7	68.9
6	flake	crushed platform	feather	left	1	-	2	-	25.5	43.6	41.1	9.7	25.5
7	flake	-	feather	orthogonal	7	1	3	5.2	17.4	39.1	40.6	13.3	16.6
8	flake	-	feather	proximal	2	0	3	4.8	34.7	47	37.8	26.8	39.9
9	flake	-	feather	proximal	0	1	3	8.4	-	40.5	35.3	11	12.7
10	flake	snap	-	proximal	1	0	2	3.7	-	41.7	39.4	13.6	17.5
11	flake	snap	feather	proximal	1	2	3	5.6	25.3	34.4	38.4	8.4	10.3
12	core	Shatter	-	proximal	3	0	3	-	45.3	55.6	60.9	54.3	195.8
13	flake	-	feather	proximal	2	2	2	3.6	25.4	25.4	34.5	5.5	3.6
14	flake	chipping	feather	proximal	2	0	2	8	26.2	31.3	25.5	8.7	6.3
15	flake	-	hinge	proximal	2	1	1	9.1	29.3	29.3	32.6	10.4	8.4
16	flake	-	feather	proximal	7	1	1	5.9	29.6	31.4	21	6.6	3.7
17	flake	snap	-	proximal	2	0	2	3.5	23.5	23.5	28.1	5.3	3.9
18	flake	-	feather	proximal	4	1	2	2.3	55.1	57.5	36.2	9.0	18.6

Supplementary Information Figures

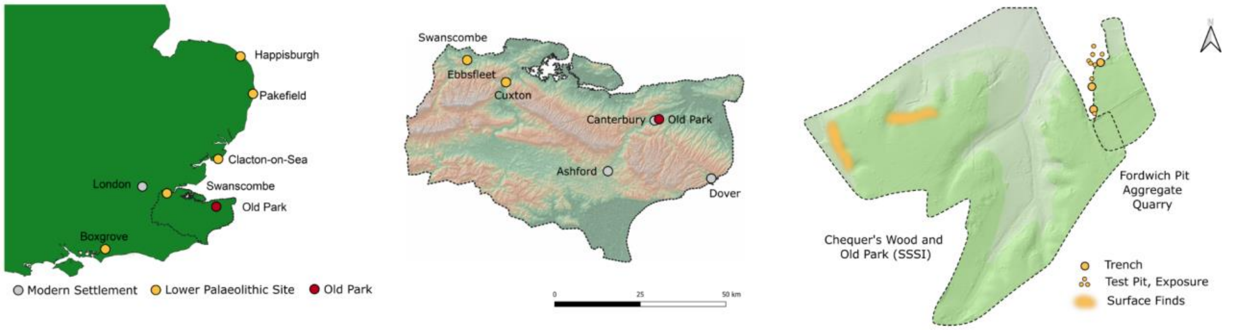

Supplementary Figure 1: Maps depicting the location of Old Park within Britain (top left), Kent (top centre) and relative to Fordwich Pit (top right). Note that the location of artefact finds, trench, test trenches and exposures are noted in this version but were not able to be included in the main Figure 1 due to a request from a relevant external party. Trenches 1, 2 and 3 are identifiable in Supplementary Figure 2.

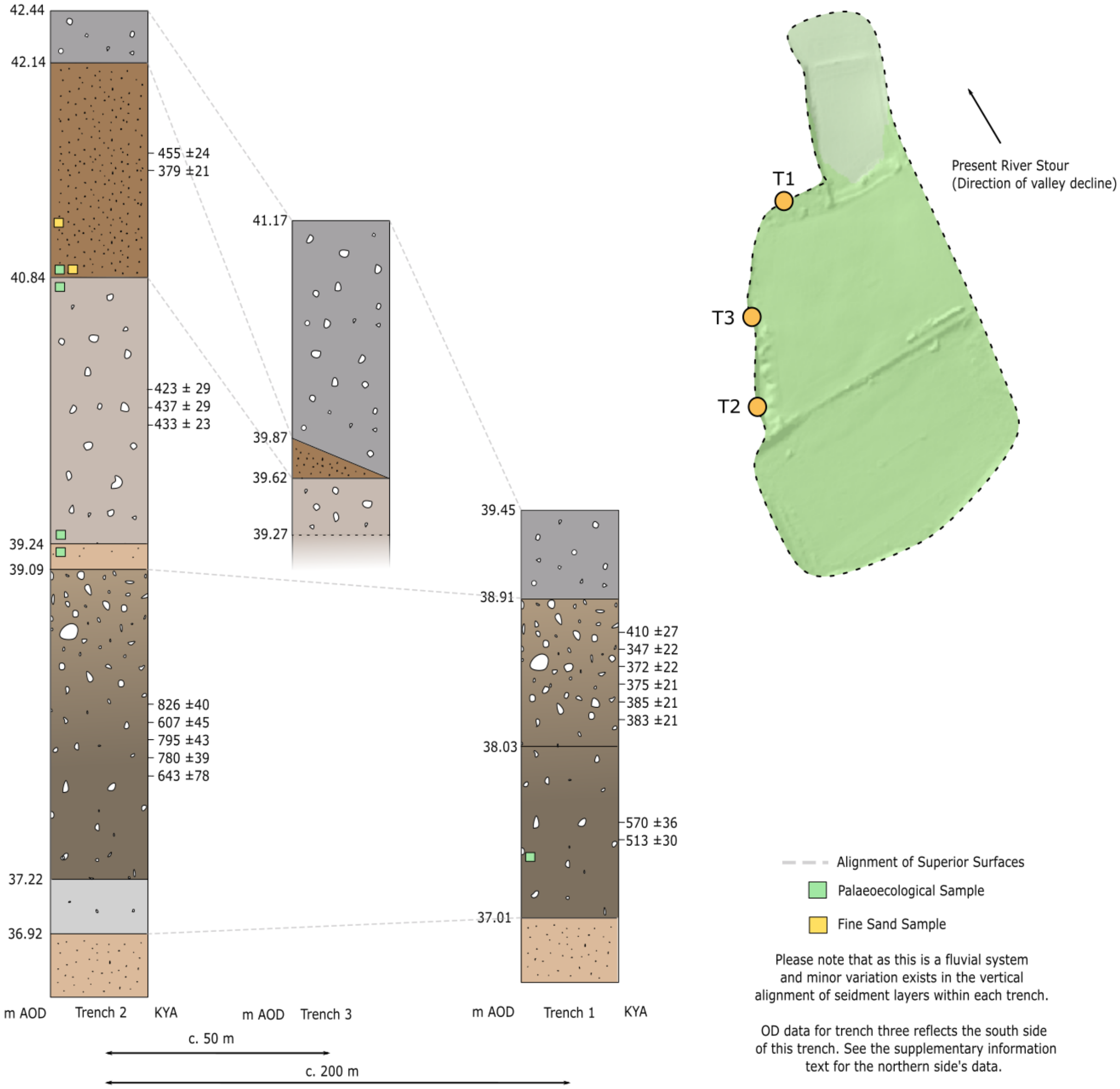

Supplementary Figure 2: The location of the palaeoecological, IR-RF samples and artefacts from within the stratigraphy of all three trenches. In the top right is our interpretation of the overarching stratigraphy, as highlighted in Key et al. (2022). In the bottom right is are the positions of the three trenches around the edge of the historically worked quarry.

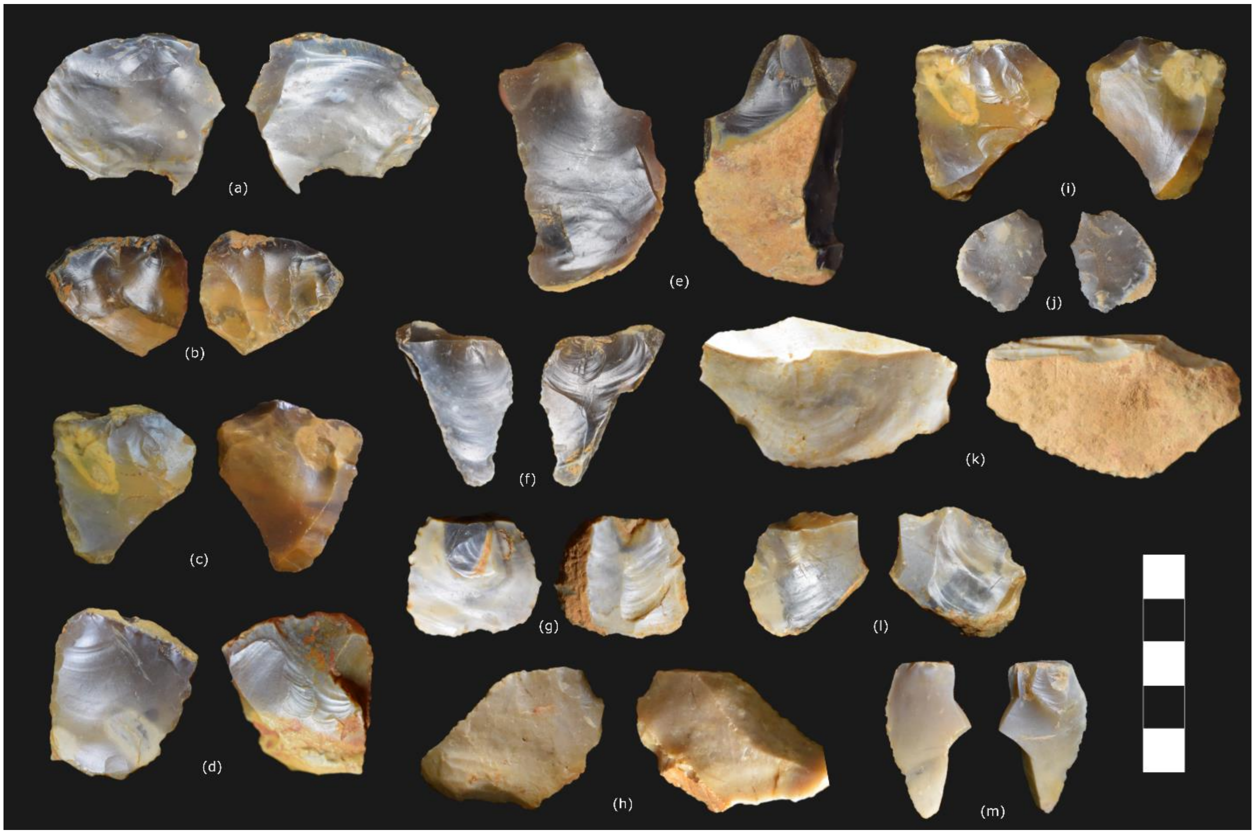

Supplementary Figure 3: A selection of flakes from Trench One recovered after those reported in Key et al. (2022) (i.e., the 2021 – 2023 field seasons). These represent artefacts assigned with absolute or high security in their anthropogenic origin. Please see Supplementary Figure 4 for examples of lithic objects from Trench One that may or may not represent flakes removed by hominins. The intention of including Supplementary Figures 3 *and* 4 is to allow direct comparison, emphasise the difficulties that can be faced when identifying stone artefacts within fluvial contexts, but also the clear differences between flakes assigned as human-made with confidence and those that are more difficult to discern.

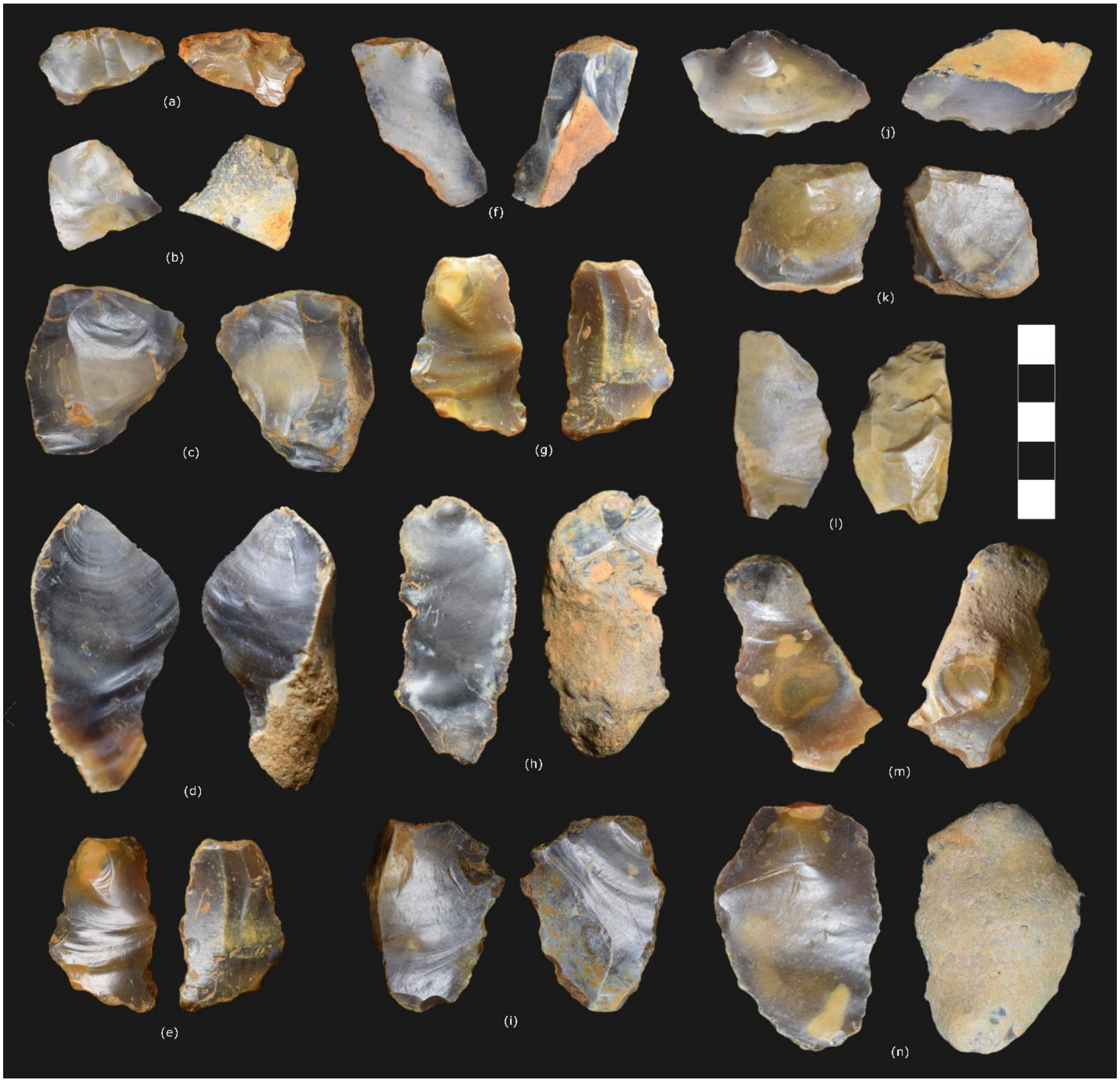

Supplementary Figure 4: A selection of lithic objects from Trench One recovered after those reported in Key et al. (2022). These represent a series of items displaying some features indicative of having been produced by hominins, but due to an absence of all required features, or having been heavily abraded within the river system, it is impossible to assign their anthropogenic origin with security. Items (g), (e), (i), and (l) are classic examples of heavily rolled and abraded lithic objects that could very easily be flake stone tools, be it due to the presence of dorsal stepping and flake scars, clear ventral surfaces with ripples and errailure scaring, or (seemingly) points of impact, but their preservation prevents assignment with any security. Item (d) is an interesting example as in any other context it would be a clear flake, and potentially it is, but its exceptionally fresh preservation raises some questions about whether it was created during post deposition movement of the sediment. Items (b), (m), (n), and (j) all display dorsal surfaces displaying a high percentage of cortex, substantially increasing the likelihood of their natural origin as there is no evidence of flake removals prior to these objects being detached. Nonetheless, their ventral surfaces and/or size indicate some features suggestive of having been human-made.

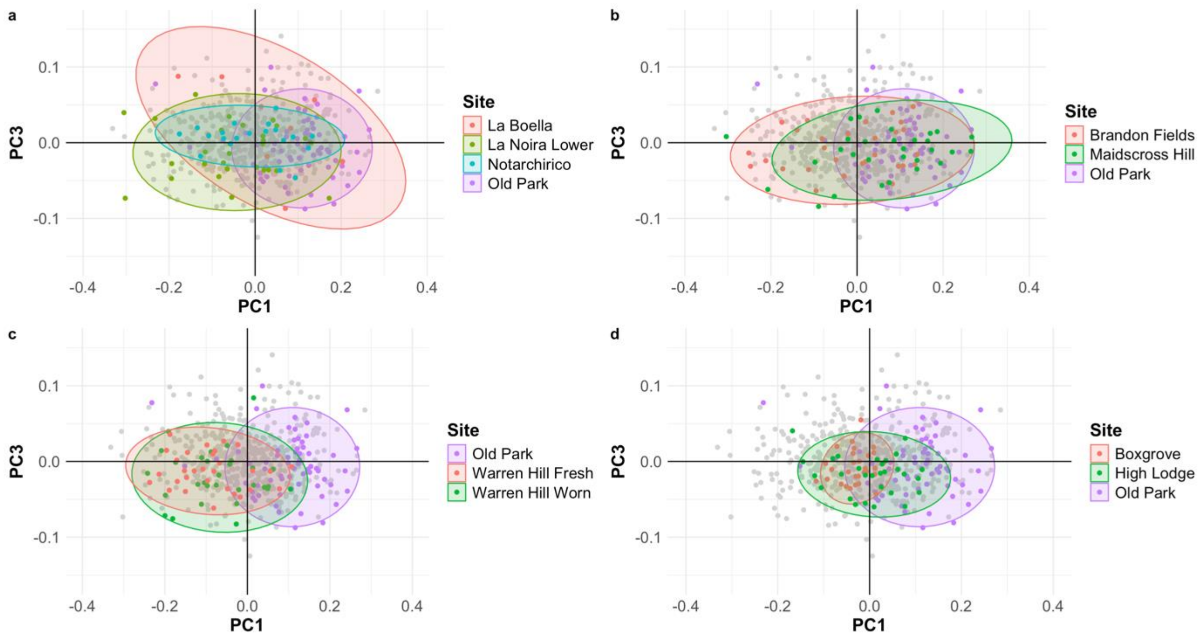

Supplementary Figure 5: 2D shape of handaxes from Old Park relative to other European assemblages. a = Plotted with very early European assemblages, b = Plotted with early British assemblages, c = Plotted with artefacts from Warren Hill, and d = Plotted with artefacts from MIS13 Britain.

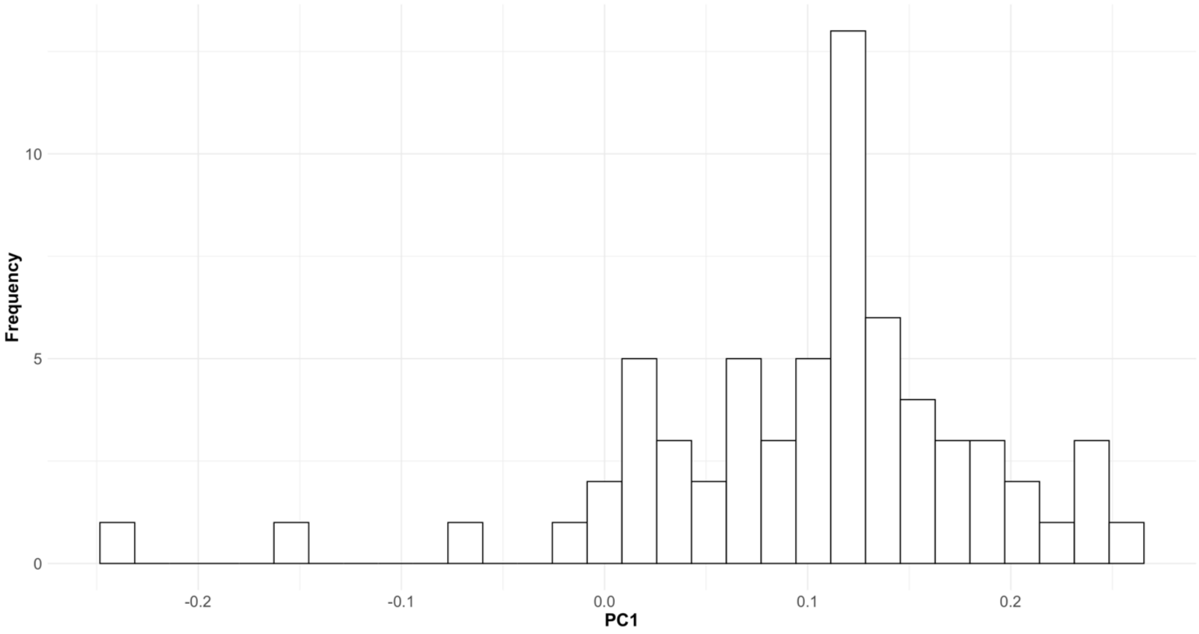

Supplementary Figure 6: Histogram of PC1 scores among the Old Park assemblage.

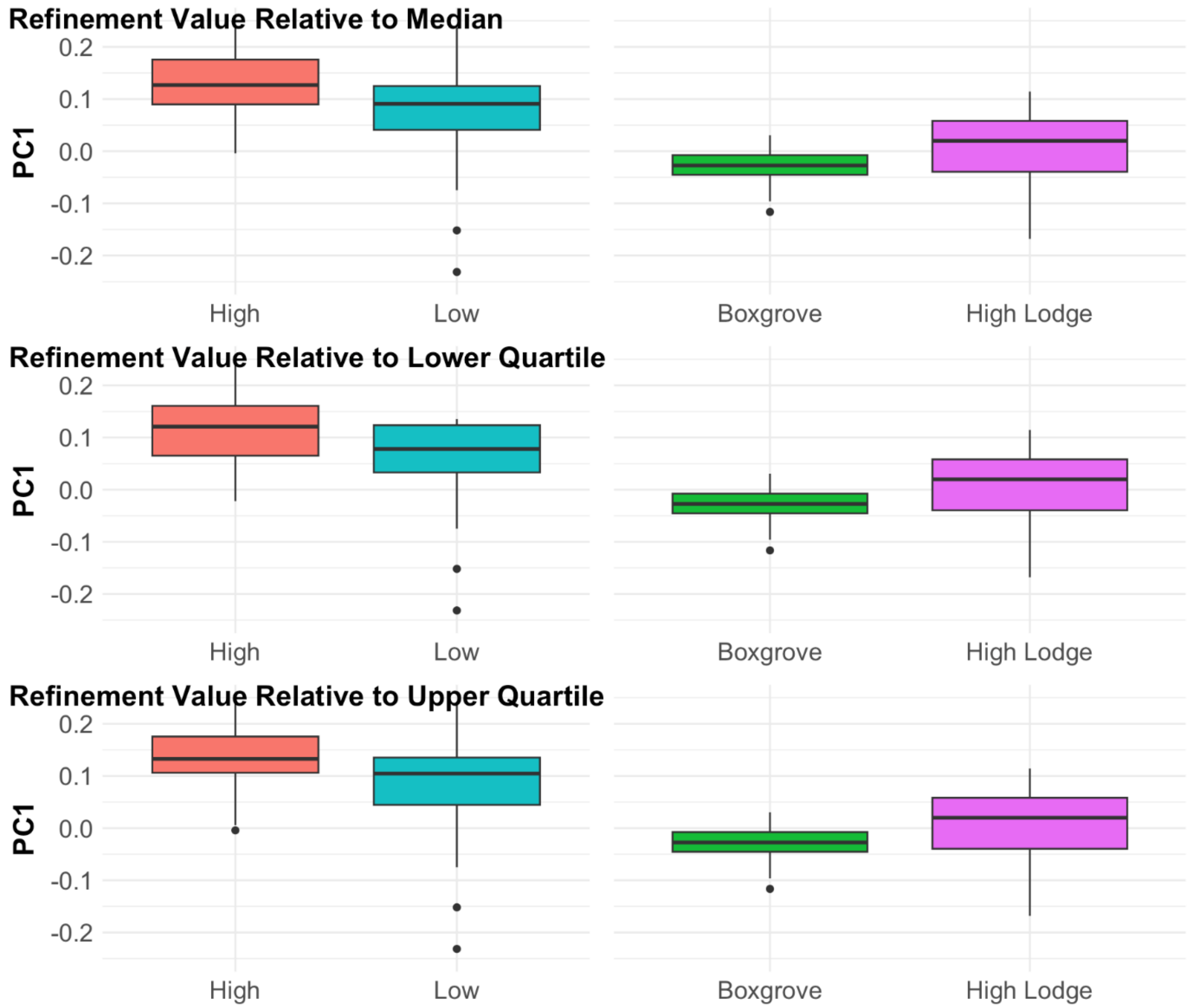

Supplementary Figure 7: PC1 of Old Park handaxes, divided above and below the Median, Lower Quartile, and Upper Quartile of refinement values. The PC1 values of MIS13 handaxes from Boxgrove and High Lodge are shown for reference. Higher refinement values (meaning thicker artefacts relative to width) relative to each marker are shown in red, with those lower than the marker (meaning thinner relative to width) shown in blue. Mean (line), upper and lower quartiles (box), and outliers (dots) are noted.
